# Exploiting GRK2 Inhibition as a Therapeutic Option in Experimental Cancer Treatment: Role of p53-Induced Mitochondrial Apoptosis

**DOI:** 10.3390/cancers12123530

**Published:** 2020-11-26

**Authors:** Jessica Gambardella, Antonella Fiordelisi, Gaetano Santulli, Michele Ciccarelli, Federica Andrea Cerasuolo, Marina Sala, Eduardo Sommella, Pietro Campiglia, Maddalena Illario, Guido Iaccarino, Daniela Sorriento

**Affiliations:** 1 Department of Advanced Biomedical Sciences, Federico II University, Via Pansini 5, 80131 Napoli, Italy; jessica.gambardella@einsteinmed.org (J.G.); antonella.fiordelisi@gmail.com (A.F.); gaetano.santulli@unina.it (G.S.); f.andrea_cerasuolo@hotmail.it (F.A.C.); 2 Department of Medicine, Albert Einstein College of Medicine, New York, NY 10461, USA; 3 Department of Medicine, Surgery and Dentistry “Scuola Medica Salernitana”, University of Salerno, 84081 Baronissi, Italy; mciccarelli@unisa.it; 4 Department of Pharmacy, University of Salerno, 84084 Fisciano, Italy; msala@unisa.it (M.S.); esommella@unisa.it (E.S.); pcampigl@unisa.it (P.C.); 5 Department of Public Health, Federico II University, 80131 Napoli, Italy; illario@unina.it

**Keywords:** GRK2, p53, thyroid cancer, mitochondrial apoptosis, GRK2 inhibition

## Abstract

**Simple Summary:**

The involvement of GRK2 in cancer growth and an inverse correlation with p53 levels were suggested in breast cancer. Furthermore, increased GRK2 expression and activity were detected in thyroid cancer, but its effects and mechanisms of action were not investigated yet. This study aimed to explore the role of GRK2 in thyroid cancer both in vitro and in vivo and its crosstalk with p53. We demonstrated that thyroid cancer cells bearing a mutant form of p53 but not p53 null cells rely on GRK2 as a mechanism of proliferation by regulating p53 levels. Indeed, GRK2 indirectly induces p53 degradation through means of its catalytic activity. The pharmacological inhibition of the kinase effectively inhibits cancer growth by inducing p53-dependent mitochondrial pathways of apoptosis. Our results demonstrate a p53-dependent effect of GRK2 in cancer and suggest kinase inhibition as a potential therapeutic strategy for thyroid cancer.

**Abstract:**

The involvement of GRK2 in cancer cell proliferation and its counter-regulation of p53 have been suggested in breast cancer even if the underlying mechanism has not yet been elucidated. Furthermore, the possibility to pharmacologically inhibit GRK2 to delay cancer cell proliferation has never been explored. We investigated this possibility by setting up a study that combined in vitro and in vivo models to underpin the crosstalk between GRK2 and p53. To reach this aim, we took advantage of the different expression of p53 in cell lines of thyroid cancer (BHT 101 expressing p53 and FRO cells, which are p53-null) in which we overexpressed or silenced GRK2. The pharmacological inhibition of GRK2 was achieved using the specific inhibitor KRX-C7. The in vivo study was performed in Balb/c nude mice, where we treated BHT-101 or FRO-derived tumors with KRX-C7. In our in vitro model, FRO cells were unaffected by GRK2 expression levels, whereas BHT-101 cells were sensitive, thus suggesting a role for p53. The regulation of p53 by GRK2 is due to phosphorylative events in Thr-55, which induce the degradation of p53. In BHT-101 cells, the pharmacologic inhibition of GRK2 by KRX-C7 increased p53 levels and activated apoptosis through the mitochondrial release of cytochrome c. These KRX-C7-mediated events were also confirmed in cancer allograft models in nude mice. In conclusion, our data showed that GRK2 counter-regulates p53 expression in cancer cells through a kinase-dependent activity. Our results further corroborate the anti-proliferative role of GRK2 inhibitors in p53-sensitive tumors and propose GRK2 as a therapeutic target in selected cancers.

## 1. Introduction

G protein-coupled receptor kinase (GRKs) family plays a key role in the regulation of cancer growth [1]. GRK5, for instance, exerts a double effect in cancer either as an inhibitor through the desensitization of G Protein coupled Receptors (GPCR) and non-GPCR receptors (TSH, PGE2R, PDGFR) or a trigger through the regulation of non-receptor substrates (p53, AUKA, and NPM1) [2]. Recently, GRK2 has also been emerging as a potential oncomodulator. Indeed, in the past decades, the GRK2 interactome was greatly explored and novel substrates have been identified that can participate in cancer progression [3]. GRK2 affects endothelial cell proliferation and migration to regulate tumor angiogenesis [4,5]. Furthermore, the overexpression of GRK2 promotes breast tumor growth in mice, while its down-regulation exerts the opposite effect and sensitizes cells to treatments [6]. In breast cancer cells, GRK2 phosphorylates HDAC6, leading to tumor growth [6]. Furthermore, it has been suggested that p53 can be a potential substrate of GRK2 [6]. GRK2 levels inversely correlate with p53-dependent responses, although a clear explanation of such an effect is missing [6,7]. The finding that the kinase participates in tumor growth comes with the corollary that kinase inhibition could be a potential strategy to delay tumor growth [8,9].

It is known that p53 is an oncosuppressor that regulates cancer development in a transcriptionally dependent and independent manner [10]. Indeed, p53 not only regulates the transcription of anti-cancer genes in the nucleus, but also directly triggers the apoptotic pathway in mitochondria [11,12]. Several tumors, including thyroid tumors, differ in p53 expression and genetic profile, showing a high incidence of mutations in the DNA-binding domain leading to the loss of p53 transcriptional activity [13]. We have recently demonstrated that inhibitors of the MDM2-dependent degradation of p53 exert anti-cancer effects also in the cells carrying these mutations [11], suggesting that in the absence of the transcriptional activity, the mitochondrial apoptotic pathway induced by p53 is still active [11]. Based on such a background, we evaluated the molecular mechanism by which GRK2 affects p53 signaling and we tested the effectiveness of GRK2 inhibition on cancer growth. Given the recurrence of p53 mutation and the increased GRK2 expression and activity in thyroid carcinomas, we explored the feasibility of our hypothesis in a p53-mutated thyroid cancer cell line (BHT-101) using a p53-null cell line (FRO) as a control.

## 2. Methods

### 2.1. Cell Culture

BHT101, which expresses a p53 mutant at codon 251 (A*T*C→A*C*C; Ile→Thr), and FRO cells, which do not express p53 at all, were a kind gift of Prof Massimo Santoro (Dipartimento di Medicina molecolare e Biotecnologie Mediche—Università Federico II di Napoli, Italy). Cells were cultured in the DMEM (Gibco) supplemented with 10% v/v FBS (GE Lifesciences) and 1% penicillin–streptomycin (Gibco) at 37 °C in 95% air/5% CO_2_.

### 2.2. GRK2 Overexpression and Silencing

Cell transfection was performed with a lipo-transfection reagent (Lipofectamine 2000, Invitrogen, Carlsbad, CA, USA, #11668-019) using 2 µg of pcDNA3.1-GRK2, pcDNA3.1-GRK2-DN, or empty pcDNA3.1 plasmid according to the manufacturer’s instructions. After 36 h from transfection, GRK2 overexpression was detected by Western blotting using a specific antibody (Santa Cruz Biotechnology, Dallas, TX, USA). For GRK2 silencing, we used a pool of four target-specific 19–25-nt small interfering RNA (siRNA) provided by Santa Cruz Biotechnology (#sc-29337) together with control siRNAs represented by scrambled sequences that would not lead to the specific degradation of any known cellular mRNA. BHT cells were transfected with 100 nM of GRK2 siRNAs or control siRNAs using Lipofectamine 2000 and GRK2 expression was detected starting 24 h from transfection by Western blotting.

### 2.3. Endoplasmic Reticulum Extracts

The cellular pellet was suspended in a hypotonic extraction buffer (10 mM 4-(2-hydroxyethyl)-1-piperazineethanesulfonic acid (HEPES), pH 7.8, 25 mM KCl, 1 mM ethylene glycol-bis(β-aminoethyl ether)-N,N,N’,N’-tetraacetic acid (EGTA)) and incubated at 4 °C for 20 min to allow the cells to swell. After centrifugation at 600× *g* for 5 min, the generated pellet was suspended in an isotonic extraction buffer (10 mM HEPES, pH 7.8, 250 mM sucrose, 25 mM KCl, and 1 mM EGTA) and lyzed using a Dounce homogenizer. The homogenate was centrifuged at 1000× *g* for 10 min at 4 °C, and after aspiration of the thin floating lipid layer, the supernatant was centrifuged at 12,000× *g* for 15 min. The generated supernatant is the post-mitochondrial fraction (PMF) which contains microsomes. Calcium chloride solution equivalent to 7.5 times the volume of PMF (8 mM) was added and incubated for 15 min at 4 °C. After centrifugation at 8000× *g* for 10 min, the generated pellet containing endoplasmic reticulum- microsomes were lysed in an isotonic buffer by pipetting several times and incubating at 4 °C for 15 min. The resultant protein extract was quantified using the Bradford reagent (BioRad) and analyzed by Western blotting. The data were analyzed and the results are shown in the graph as the mean of three independent experiments.

### 2.4. Immunoprecipitation and Western Blotting

BHT-1 and FRO cells were treated with KRX-C7 for one hour and cells were collected to perform total, cytosolic, and ER extracts. Total and cytosolic lysates were prepared as described previously [14]. Immunoprecipitation and Western blot analysis were performed as previously described [11,15]. Briefly, cells were lyzed in a radioimmunoprecipitation assay buffer (RIPA)/SDS buffer (50 mM Tris–HCl (pH 7.5), 150 mM NaCl, 1% Nonidet P-40, 0.25% deoxycholate, 9.4 mg/50 mL sodium orthovanadate, 20% SDS). Protein concentration was determined by using the Pierce bicinchoninic acid (BCA) assay kit (Thermo Fisher Scientific, Waltham, MA, USA, #23225) and starting from the same protein concentration, endogenous p53 from total extracts were immunoprecipitated adding the specific antibody (cell signaling) and protein A/G agarose (Santa Cruz, #sc-2003) with 4 °C overnight incubation. After centrifugation at 5000× *g* for 5 min and extensive washes, the immunocomplexes were isolated; through the addition of 2% SDS and incubation at 95 °C, denaturation of immunocomplexes was performed that allowed removing the agarose beads. The samples were loaded onto the SDS-PAGE gel for the immunoblotting analysis and the levels of p53 phosphorylation were detected using the specific antibody (Thr55, p-p53, Santa Cruz), as well as the levels of co-immunoprecipitated GRK2 and endoplasmic reticulum Ca^2+^ ATPase (SERCA) were detected using the specific antibody from Santa Cruz and Cell Signaling, respectively. The levels of immunoglobulin (Ig) were used as a loading control. The data were analyzed and the results are shown in the graph as the mean of three independent experiments. For Western blotting, the following antibodies were used: GRK2 (#sc-13143), Actina (#sc-1615), p-p53 (#sc-377567), and ERK2 (#sc-1647) from Santa Cruz Biotecnology; p53 (#2524S), SERCA (#9580), Cytochrome c (#4272S), pRb (#9308), calnexin (#2679), and p-ERK (#4370); cleaved caspase 3 from cell signaling; Ubiquitin (#ab7254) from Abcam.

### 2.5. Immunoprecipitation and Nano-LC-MS/MS Analysis

Immunoprecipitates from cells treated with DMSO and KRX-C7 were digested: 100 μL of 50 mM ammonium bicarbonate was added to each sample. The beads were vortexed gently for 1 h. The proteins were reduced with 10 mM Dithiothreitol (DTT) for 30 min, then alkylated with 20 mM Iodoacetamide (IAA) for 1 h in the dark. The enzyme/substrate ratio was 1:100. The sample was incubated at 37 °C overnight in a Thermomixer comfort (Eppendorf, Hamburg, Germany); the supernatant was then collected. After trypsin digestion, the beads were washed twice with 50 μL of 50% Acetonitrile (ACN) and the supernatants collected pooled and dried by a gentle nitrogen steam. A nanoflow ultra-high performance liquid chromatography (UHPLC) instrument (Ultimate 3000, RSLCnano Thermo Fisher Scientific, Waltham, MA, USA) was online hyphenated to a Q Exactive mass spectrometer (Thermo Fisher Scientific, Waltham, MA, USA) with a nanoelectrospray ion source (Thermo Fisher Scientific, Waltham, MA, USA). Peptides were loaded onto a C18-reversed phase column (15 cm × 75 μm ID, 3 μm, EASYspray columnThermo). Mobile phases were A—0.1% HCOOH in water, B—0.1% HCOOH canACN; the following gradients were used: 0–30% B, 0–40 min; 30–70% B, 40–45 min; 70–95% B, 45–50 min; 95% B, hold for 5 min, with a flow rate of 300 nL/min over 70 min. Each sample was analyzed using LC-MS/MS twice. MS data were acquired using a data-dependent Top10 method, with a survey scan (350–1500 m/z) using Higher-energy C-trap dissociation (HCD) fragmentation. MS1 was acquired at a resolution of 70,000 at 400 m/z, while MS/MS at 17,500. Unassigned precursor ion charge states as well as single charged species were excluded. Isolation window was set to 3 Da and normalized collision energies (NCE) of 27 were applied. Maximum ion injection times for MS and the MS/MS scans were 50 ms and 120 ms, respectively, and Automatic gain control (ACG) values were set to 3E6 and 1e5, respectively. Dynamic exclusion: 30 s.

Raw MS data were analyzed using Proteome Discoverer software version 1.6.1.16 using the SEQUEST search engine. The maximum allowed mass deviation was set to 20 ppm. Enzyme specificity was set to trypsin, two missed cleavages were allowed. Carbamidomethylcysteine was set as a fixed modification, methionine oxidation and phosphorylation of serine, threonine, and tyrosine—as variable modifications. The spectra were searched against the *Homo Sapiens* (human) sequence database from Swiss-Prot database 2020_11, taxonomy ID 9606.

### 2.6. Measurement of Mitochondrial Calcium Content

Analysis of the mitochondrial calcium content was performed as previously described [16]. Briefly, mitochondria were isolated by cells using an EGTA/EDTA-free buffer to avoid chelating Ca^2+^, and, after extensive washes, were lyzed in 0.6 M HCl and sonicated (2 × 10 s). The absolute calcium content was determined using the o-cresolphthalein complexone assay (Cayman Chemical, #701220) according to the manufacturer’s instructions. The data were analyzed and the results are shown in the graph as the mean of three independent experiments.

### 2.7. Proliferation Assay

The assay was performed as described previously [17], using a CyQUANT^®^ NF Cell Proliferation Assay Kit (Invitrogen #C35006) according to the manufacturer’s instructions. The cells were treated with KRX-C7 for 24 h. The data were analyzed and the results are shown in the graph as the mean of three independent experiments.

### 2.8. In Vivo Study

Experiments were carried out following the NIH guidelines for animal investigation in 8-week-old male BALB/c immunocompetent nude mice (weight range: 24 ± 1.2), which were purchased by Charles River. The mice were housed in the animal facility of the Department of Translational Medical Sciences of Federico II University of Naples (Italy) and health monitoring was performed according to the Federation of European Laboratory Animal Science Associations (FELASA) guidelines. All mice were fed commercial mouse diet food with a 12-h light-dark cycle under pathogen-free conditions and had access to food and water ad libitum. All in vivo experimental protocols were approved by the Federico II University’s Ethical Committee for Animal Studies and were carried out in accordance with EU Directive 2010/63/EU for animal experiments. During the experiments, the animals were carefully monitored and euthanized if they showed signs of pain, such as reduced food or water intake, skin ulcers, hunched posture, excessive weight loss. Euthanasia was performed using a mix of isoflurane and oxygen inhalation followed by cervical dislocation.

Sample size calculation showed that 5 mice/group were needed to achieve the statistical power of 0.8 based on our previous in vivo experiments [14,17]. BHT-101 and FRO cells were injected in Balb/c nude mice as described previously [14,17]. Briefly, a suspension containing 2 × 10^6^ cancer cells in 200 μL of DMEM was injected subcutaneously in the dorsal side of nude mice to induce tumor formation. The mice were randomly divided into two groups (5 mice/group) and treated with intra-tumor injections of KRX-C7 (1 mg/kg) or saline twice a week for 3 weeks. Tumor growth was measured with a caliper twice a week. After three weeks, tumors were collected for the Western blot analysis. For all the experiments, the animals were anesthetized with 3% isoflurane and euthanasia was performed by cervical dislocation.

### 2.9. Statistical Analysis

All values are presented as mean ± SEM. Statistical analysis was performed using the Student’s t-test (for 2 groups) and two-way ANOVA to compare the different parameters between the different groups. The significance level of *p* < 0.05 was assumed for all statistical evaluations. The statistical data were computed with the GraphPad Prism software version 8.0 for Mac (GraphPad Software, San Diego, CA, USA).

## 3. Results

### 3.1. GRK2 Induces Cancer Cell Proliferation by Regulating p53

**Figure 1:**
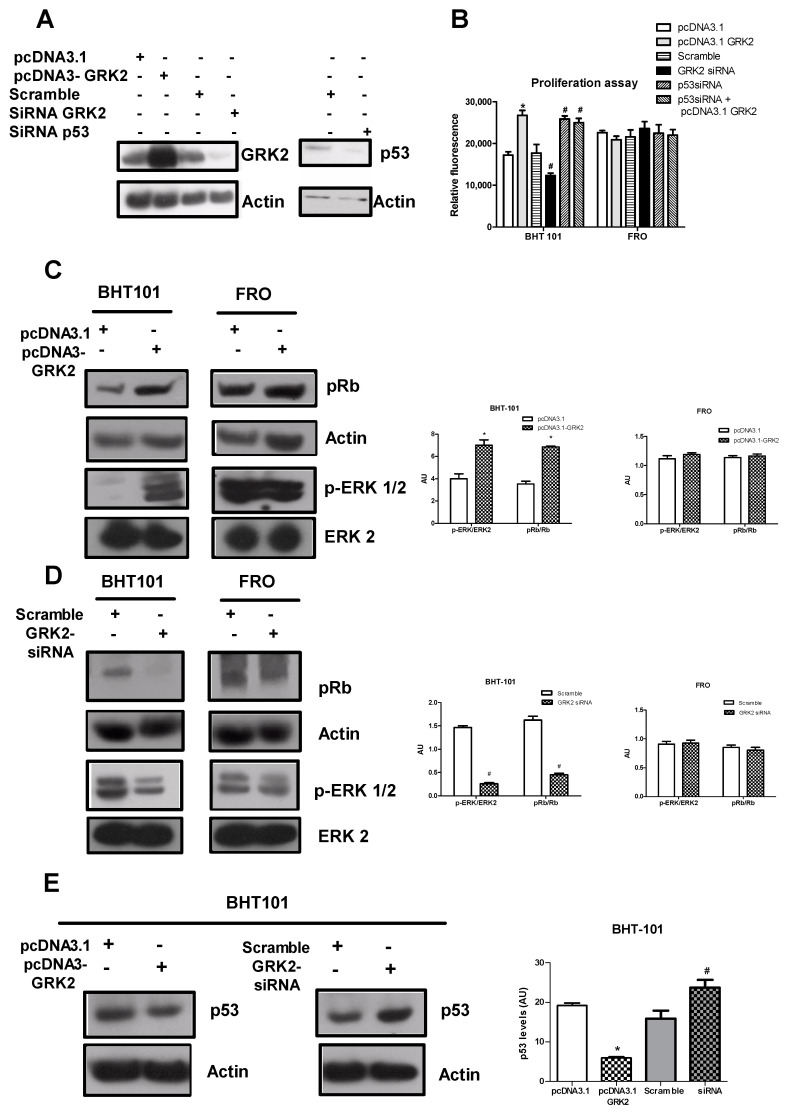
GRK2 induces cancer cell proliferation by regulating p53. (**A**) GRK2 and p53 silencing were induced by transient transfection in BHT-101 cells. Protein levels were evaluated by the Western blot analysis. Actin was used as a loading control. (**B**) BHT-101 and FRO cells were transfected with pcDNA3.1, pcDNA3.1-GRK2, Scramble, GRK2 siRNA, or p53 siRNA as indicated, and cell proliferation was evaluated. The overexpression of GRK2 and p53 silencing induces BHT-101 cell proliferation, while GRK2 silencing exerts an opposite effect, but no effects were detected in FRO cells in any condition. Densitometric analysis is shown in the bar graph; * *p* < 0.05 vs. pcDNA3.1 and # *p* < 0.05 vs. Scramble. (**C**,**D**) The phosphorylated forms of ERK and Rb were evaluated by Western blotting. Both are increased by GRK2 overexpression (**C**) and inhibited by its silencing in BHT-101 cells (**D**), but are unchanged in FRO cells. Actin and ERK2 were used as a loading control of pRb and *p*-ERK2, respectively. Densitometric analysis is shown in the bar graph; * *p* < 0.05 vs. pcDNA3.1 and # *p* < 0.05 vs. Scramble. (**E**) Western blotting was used to detect p53 levels. In BHT-101 cells, the overexpression of GRK2 reduces p53 levels, while its silencing exerts the opposite effect. Actin was used as a loading control. Densitometric analysis is shown in the bar graph; * *p* < 0.05 vs. pcDNA3.1 and # *p* < 0.05 vs. Scramble.

We evaluated the effects of GRK2 overexpression and silencing in two thyroid cancer cell lines, BHT-101 and FRO cells, which differ in p53 but not GRK2 expression. Figure 1A shows transgenic GRK2 overexpression and GRK2 and p53 silencing in BHT-101 cells. The overexpression and silencing of GRK2 in BHT-101 cells exert opposite effects on cell proliferation (Figure 1B), while in FRO cells, cell proliferation was insensitive to the different rates of GRK2 (Figure 1B). Accordingly, P-ERK and pRb levels, markers of proliferation, are increased by GRK2 overexpression (Figure 1C) and inhibited by its silencing in BHT-101 cells (Figure 1D). However, their levels are unchanged in FRO cells (Figure 1C,D). These data suggest that p53 expression could be involved in GRK2-dependent proliferation of cancer cells. To assess this issue, we silenced p53 in BHT-101 cells and evaluated the effect on cell proliferation. Silencing of p53 significantly increased cell proliferation both basally and in response to GRK2 overexpression (Figure 1B), confirming the key role of p53 in GRK2-dependent cancer cell proliferation. In line with previous findings in breast cancer cells [6,7], in BHT-101 cells, p53 levels were inversely correlated with the GRK2 expression rate. Indeed, the overexpression of the kinase reduced p53 levels, while its silencing exerted the opposite effect (Figure 1E). Altogether, these results suggest that p53 is a possible mediator for GRK2 regulation of cancer cell proliferation.

### 3.2. GRK2 Inhibits p53 Signaling through Its Catalytic Activity

**Figure 2:**
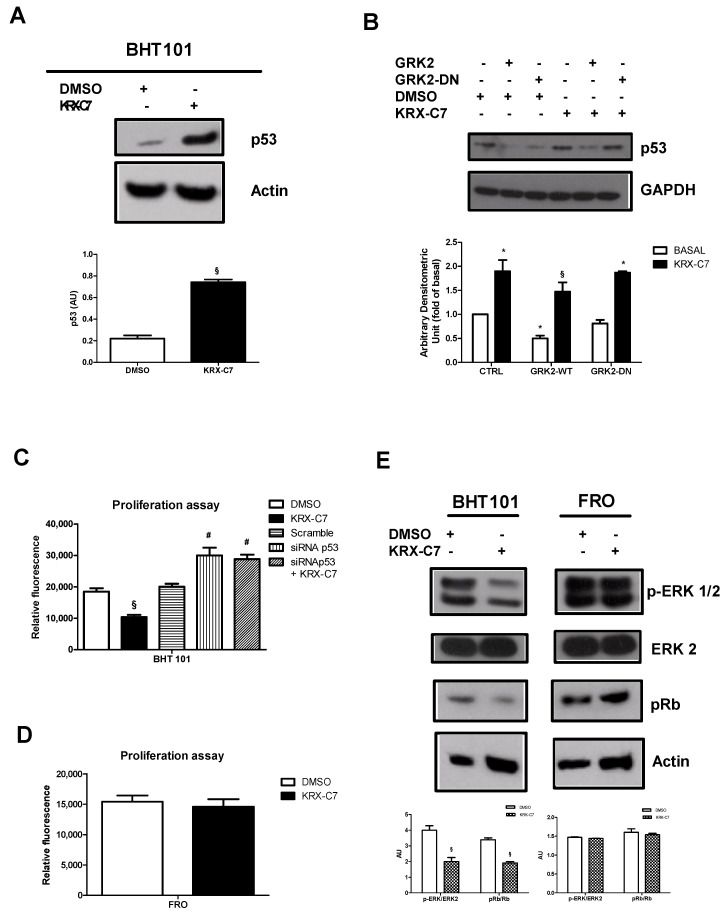
GRK2 inhibits p53 signaling through its catalytic activity. (**A**) BHT-101 cells were treated with KRX-C7 and p53 levels were evaluated by Western blotting. Densitometric analysis is shown in the bar graph, * *p* < 0.05 vs. the control and § *p* < 0.05 vs. KRX-C7. (**B**) BHT-101 and FRO cells were transfected with GRK2 and GRK2-DN plasmids and p53 levels were evaluated by Western blotting both basally and in response to KRX-C7. Densitometric analysis is shown in the bar graph, * *p* < 0.05 vs. the control and § *p* < 0.05 vs. KRX-C7. (**C**,**D**) BHT-101 cells were treated with DMSO or KRX-C7 after the silencing of p53 and cell proliferation was evaluated. KRX-C7 inhibited BHT-101 proliferation, but was not more effective in cells with p53 silencing (**C**). KRX-C7 was not effective in FRO cells (**D**). Densitometric analysis is shown in the bar graph; § *p* < 0.05 vs. DMSO and # *p* < 0.05 vs. Scramble. (**E**) The phosphorylated forms of ERK and Rb were evaluated by Western blotting. KRX-C7 inhibited the phosphorylation of ERK and Rb in BHT-101 cells, but not in FRO cells. Densitometric analysis is shown in the bar graph; § *p* < 0.05 vs. DMSO.

It has been suggested that GRK2 regulates breast cancer growth through catalytic activity: hence, we evaluated whether GRK2 inhibition could affect p53 responses. Kinase inhibition was induced by KRX-C7, a specific inhibitor that we synthesized and tested in previous studies [18,19]. KRX-C7 increased p53 levels (Figure 2A), suggesting that the catalytic activity of GRK2 was needed to induce cancer cell proliferation through the down-regulation of p53. This finding was further confirmed by overexpressing the kinase-dead mutant of GRK2 (GRK2-DN). Indeed, GRK2-DN did not affect p53 levels in response to KRX-C7 compared with the wild-type form (Figure 2B). In BHT-101 cells, KRX-C7 also blocked cell proliferation (Figure 2C) and reduced the phosphorylation of both ERK and Rb (Figure 2E), but was not effective in FRO cells (Figure 2D,E) and in BHT-101 cells in response to p53 silencing (Figure 2C). According to the data observed in BHT-101 cells, KRX-C7 was also effective in reducing the proliferation of another thyroid cancer cell line bearing a different mutation of p53 (Figure S1).

### 3.3. GRK2 Regulates p53 Levels by Interfering with Its Degradation

**Figure 3:**
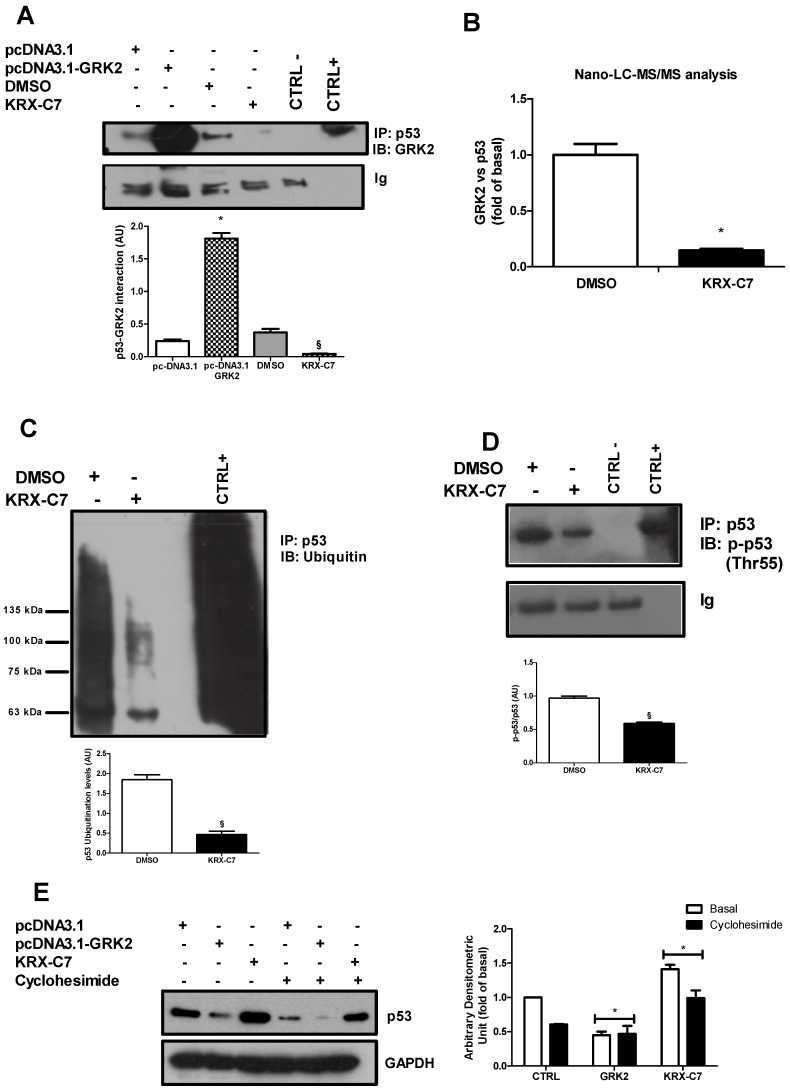
GRK2 regulates p53 levels by interfering with its degradation. (**A**) BHT-101 cells were transfected with pcDNA3.1/pcDNA3.1–GRK2 or treated with DMSO/KRX-C7. GRK2 was detected by Western blotting after p53 was immunoprecipitated from whole extracts. The whole lysate from control cells was used as the positive control. As the negative control, the assay was performed using a non-specific antibody from the same species as the immunoprecipitation (IP) antibody. GRK2 precipitated p53 and such interaction is higher in response to GRK2 overexpression, but is reduced in response to KRX-C7. IP = immunoprecipitated, IB = immunoblotting. Densitometric analysis is shown in the bar graph; § *p* < 0.05 vs. DMSO and * *p* < 0.05 vs. pc-DNA3.1. (**B**) Cells were treated with DMSO or KRX-C7 and p53 was immunoprecipitated from whole-cell extracts. Immunoprecipitates were analyzed by nano-LC-MS/MS to assess the presence of GRK2 and p53 and their interaction. The results are shown in the the bar graph; * *p* < 0.05 vs. DMSO. (**C**) Western blotting was performed using an anti-ubiquitin antibody after p53 was immunoprecipitated from total lysates of the BHT-101 cells treated with DMSO or KRX-C7. KRX-C7 reduced p53 ubiquitination. The results are shown in the bar graph; § *p* < 0.05 vs. DMSO. (**D**) Western blotting was performed to detect the phosphorylated form of p53 in threonine 55 after p53 was immunoprecipitated from total lysates of the BHT-101 cells treated with DMSO or KRX-C7. The whole lysate was used as the positive control. As the negative control, the assay was performed using a non-specific antibody from the same species as the IP antibody. KRX-C7 reduced p53 phosphorylation. Densitometric analysis is shown in the bar graph; § *p* < 0.05 vs. DMSO. (**E**) BHT-101 cells were pretreated for 30 min with cycloheximide (50 μg/mL). Western blotting was used to evaluate p53 levels in response to GRK2 overexpression and KRX-C7. Treatments were also effective in modulating the p53 half-life in the presence of cycloheximide. Densitometric analysis is shown in the bar graph; * *p* < 0.05 vs. the control.

We explored the mechanism through which GRK2 rapidly modifies p53 levels. To this aim, we evaluated whether GRK2 was able to bind and phosphorylate p53 and the possible effects on p53 degradation. Our results show that GRK2 was basally able to precipitate p53 and such interaction was higher in response to GRK2 overexpression, but was reduced in response to KRX-C7 (Figure 3A). To further confirm the effect of KRX-C7 on the disruption of GRK2 and p53 interaction, we performed the nano-LC-MS/MS analysis and corrected the GRK2/p53 rate in the same sample. The results confirmed the interaction between GRK2 and p53 that was reduced in response to KRX-C7 (Figure 3B). We then explored the possibility that GRK2 could regulate p53 levels by interfering with its degradation and showed that KRX-C7 reduced p53 ubiquitination (Figure 3C). Since p53 proteasomal degradation depends on Thr55 phosphorylation [20], we evaluated whether GRK2 affected such an event. As expected, KRX-C7 reduced p53 phosphorylation in threonine 55 (Figure 3D) in line with its effects on p53 degradation. Altogether, these findings suggest that GRK2 regulates p53 levels by inducing its degradation in a kinase-dependent manner. To confirm these findings, we evaluated the effects of GRK2 overexpression and inhibition on p53 levels in the BHT-101 cells pretreated with cycloheximide to inhibit de novo protein synthesis. Figure 3E shows that GRK2 overexpression and KRX-C7 also mod ulate p53 half-life in response to the inhibition of protein synthesis.

### 3.4. KRX-C7 Induces p53 Mitochondrial Pathway of Apoptosis

**Figure 4:**
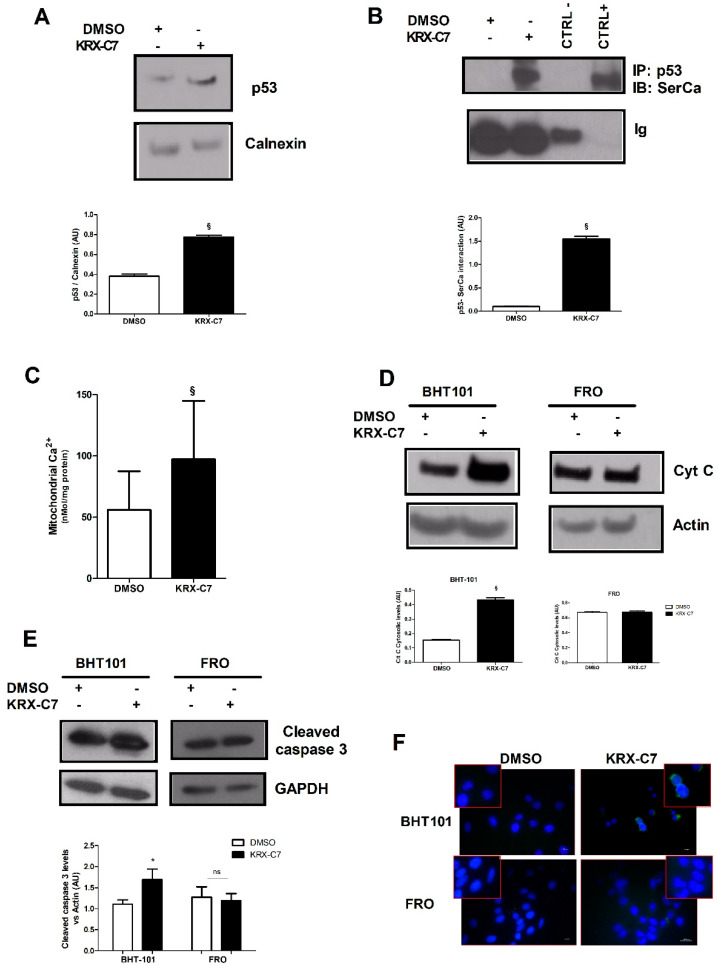
KRX-C7 induces p53 mitochondrial pathway of apoptosis. (**A**) Western blotting was used to detect p53 levels in the endoplasmic reticulum extracts isolated from the BHT-101 cells treated with DMSO or KRX-C7. The treatment with KRX-C7 increased p53 levels in such extracts. Densitometric analysis is shown in the bar graph; § *p* < 0.05 vs. DMSO. (**B**) Whole extracts of the BHT-101 cells treated with DMSO or KRX-C7 were used to immunoprecipitate p53, and SERCA was detected by Western blotting. The whole lysate was used as the positive control. As the negative control, the assay was performed using a non-specific antibody from the same species as the IP antibody. KRX-C7 increased the interaction between p53 and SERCA on the reticulum. Densitometric analysis is shown in the bar graph; § *p* < 0.05 vs. DMSO. (**C**) The absolute calcium content was determined using the o-cresolphthalein complexone assay in the mitochondria isolated from the BHT-101 cells treated with DMSO or KRX-C7. KRX-C7 increased mitochondrial calcium content. Densitometric analysis is shown in the bar graph; § *p* < 0.05 vs. DMSO. (**D**) Cytochrome c release from mitochondria was evaluated by Western blotting in cytosolic extracts from the BHT-101 and FRO cells treated with DMSO or KRX-C7. KRX-C7 increased cytochrome c levels in BHT-101, but not in FRO cells. Densitometric analysis is shown in the bar graph; § *p* < 0.05 vs. DMSO. (**E**,**F**) Apoptosis was evaluated in BHT-101 and FRO cells using Annexin V staining (**F**) and cleaved caspase 3 levels (**E**). Cells were treated with DMSO or KRX-C7. KRX-C7 induced apoptosis in BHT-101 cells, but was not effective in FRO cells (**F**). Accordingly, the levels of cleaved caspase 3 were increased in BHT-101 cells in response to KRX-C7 but did not change in FRO cells (**E**). ns = not significant; * *p* < 0.05 vs. DMSO.

It is known that p53 also activates apoptosis in a transcriptionally independent manner through its interaction with SERCA on the endoplasmic reticulum (ER) that regulates the calcium flux to mitochondria and the release of cytochrome c from this organelle [21]. To assess whether GRK2 could affect such a pathway, we retraced it in our experimental model. First, we explored the expression levels of p53 in response to KRX-C7 in the ER compartment finding that KRX-C7 increased p53 levels in the ER (Figure 4A); this was associated with increased interaction with SERCA (Figure 4B). These events were followed by mitochondrial calcium overload (Figure 4C) and cytochrome c release from mitochondria (Figure 4D). This latter was not observed in p53-null cells (Figure 4D). We then explored the effects of KRX-C7 on apoptosis by analyzing specific markers downstream of cytochrome c. In particular, we evaluated cleaved caspase 3 levels (Figure 4E) and Annexin V staining (Figure 4F). Both experiments confirmed the ability of KRX-C7 to induce cell apoptosis in BHT-101 (Annexin V: +35 ± 2%; cleaved caspase 3: +49 ± 1.5%; KRX-C7 versus DMSO), but not in FRO cells. These findings suggest that GRK2 inhibits the p53 mitochondrial pathway of apoptosis.

### 3.5. KRX-C7 Inhibits Tumor Growth In Vivo

**Figure 5:**
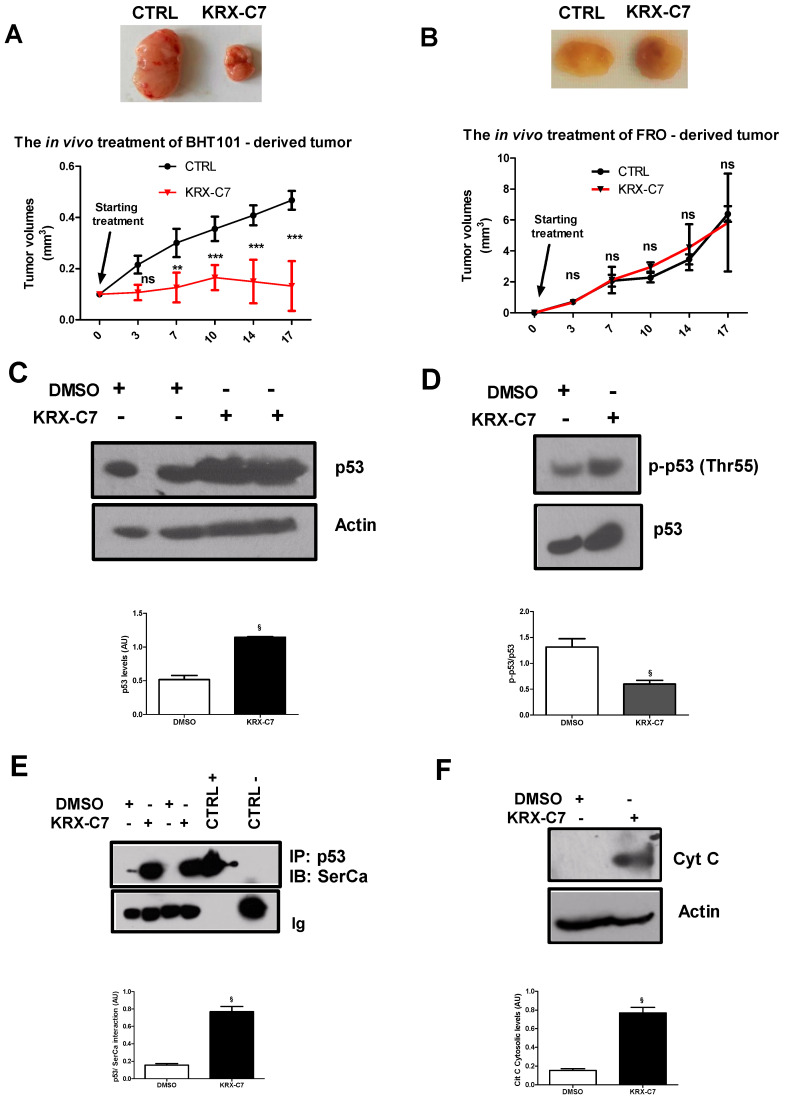
KRX-C7 inhibits tumor growth in vivo. (**A**,**B**) BHT-101 or FRO cells were subcutaneously injected to Balb/c nude mice. Treatment was performed through intra-tumor injection of DMSO or KRX-C7. Tumor volumes were measured twice a week for three weeks using a caliper. KRX-C7 was effective in reducing BHT-101-derived tumor growth (**A**), but was not effective in FRO-derived tumors (**B**). ns = not significant. Densitometric analysis is shown in the bar graph; ** *p* < 0.01, *** *p* < 0.001 vs. the starting treatment. (**C**–**F**) Tumors were collected at the end of the treatment and lyzed for measuring p53 levels and phospho-p53 (Thr55) in whole lysates, cytochrome c levels in cytosolic extracts, and the interaction between p53 and SERCA in whole extracts. In BHT-101-derived tumors, KRX-C7 induced p53 levels (**C**), inhibited its phosphorylation in threonine 55 (**D**), increased the p53/SERCA interaction (**E**), and induced cytochrome c release from mitochondria (**F**). In the immunoprecipitation assay, the whole lysate was used as the positive control and, as the negative control, the assay was performed using a non-specific antibody from the same species as the IP antibody. Densitometric analysis is shown in the bar graph; § *p* < 0.05 vs. DMSO.

To translate in vitro findings in a pre-clinical model of cancer, we evaluated the oncologic therapeutic feature of KRX-C7 in vivo in Balb/c nude mice bearing BHT-101- or FRO-derived tumors. To this aim, BHT-101 and FRO cells were injected to Balb/c nude mice as described in the methods, and the derived tumors were treated with KRX-C7. In the basal conditions, FRO-derived tumors grew more actively than BHT-101-derived tumors, which was consistent with the absence of p53. In treated tumors, KRX-C7 inhibited BHT-101-derived tumor growth in a time-dependent manner (Figure 5A), but was not effective in FRO-derived tumors (Figure 5B). Our in vivo findings suggest that GRK2 inhibition could be an effective strategy to delay tumor growth. In line with our in vitro findings, in BHT-101-derived tumors, KRX-C7 increased p53 levels (Figure 5C) by inhibiting its phosphorylation in threonine 55 (Figure 5D). This caused an increase in the p53-SERCA interaction (Figure 5E), which was associated with an up-regulation of cytochrome c release from mitochondria (Figure 5F). These findings confirm the effectiveness of KRX-C7 in reducing tumor growth by inducing the p53 mitochondrial pathway of apoptosis.

## 4. Discussion

Our study showed for the first time that GRK2 acts as a cancer activator through p53. Accordingly, our results propose the inhibition of GRK2 activity as a potential therapeutic strategy for GRK2- and p53-expressing cancers. Crosstalk between GRK2 and p53 has been previously suggested in breast cancer cells expressing the wild-type form of p53 [5]. Indeed, GRK2 protein levels inversely correlate with p53-dependent apoptotic responses [7]. Since GRK2 levels and activity are also increased in thyroid cancer tissues [9], here, we assessed such crosstalk in cancer cell lines derived from human thyroid anaplastic cancer: BHT-101, which carries a mutation in the p53 gene which inhibits DNA binding, and FRO, which is a p53-null cell line. We choose a mutated cancer cell line, since the loss of function of tumor suppressor proteins is the most frequent alteration in thyroid cancer [22,23]. In particular, loss of p53 function due to mutation in the DNA binding domain is a peculiar feature of anaplastic thyroid carcinomas which causes tumor progression and chemoresistance [24,25]. Moreover, TP53 gene inactivation seems to play a major role in the progression from differentiated to undifferentiated carcinomas, suggesting the involvement of p53 in the late stages of the carcinogenic process [26]. Indeed, this particular type of mutation is rare in differentiated thyroid cancer [23], but ranges from 50% to 80% in undifferentiated tumors [27,28].

The lack of p53 transcriptional activity does not affect the protein ability to induce apoptosis via the activation of the mitochondrial apoptotic pathway [29]. Thus, in the cancer cells characterized by loss of p53 function in the nucleus, p53 still represents a potential target for inducing cancer cell death.

GRK2 exerts a key role in the development and progression of breast cancer growth and metastatization through its catalytic activity [4,6,7]. Here, we show that this ability of GRK2 is also retained in thyroid cancer, where it is highly active [8,9]. The kinase overexpression induces BHT-101 cell proliferation which is associated with an increase in p-ERK and pRb levels. Accordingly, the silencing of GRK2 reduces such effects. This GRK2-dependent phenotype is reproduced neither in p53-null cells nor in BHT-101 cells with silencing of p53 suggesting that p53 is a fundamental GRK2 target in the regulation of cancer progression. To our knowledge, such a tight link between GRK2 and p53 in cancer had never been shown previously. We also demonstrated for the first time the mechanism by which GRK2 affects p53 signaling. GRK2 can interact with p53 and regulate its phosphorylation in threonine 55, a post-translational modification that is known to favor protein ubiquitination and degradation. Thus, GRK2 reduces p53 levels in cancer cells by regulating its degradation. Previously, Chen and colleagues demonstrated that GRK5, but not GRK2, was able to directly phosphorylate p53 [30]. In line with these findings, we did not prove that the kinase directly phosphorylates p53, but just its ability to regulate p53 phosphorylation. Thus, it is likely that GRK2 is part of a complex pathway which finally leads to p53 phosphorylation. In this context, we previously demonstrated that GRK2 directly affects mitochondrial function [31,32,33], therefore, we cannot exclude a direct mitochondrial effect of GRK2 in cancer. Further studies will be needed to clarify this issue.

Given the suggested involvement of GRK2 catalytic activity in such a pathway [6], we explored the possibility that its inhibition could effectively delay tumor growth in vivo. We inhibited GRK2 activity through a synthetic peptide reproducing the HJ loop of the kinase catalytic domain that we previously designed and tested [34]. The inhibition of GRK2 activity by KRX-C7 significantly reduces BHT-101-derived tumor growth by inducing mitochondrial p53 activity but is not effective in FRO-derived tumors. These findings confirm that GRK2 pro-tumoral activity is strictly dependent on p53, since it is based on kinase-dependent p53 phosphorylation and degradation.

In response to stresses, p53 accumulates at the endoplasmic reticulum–mitochondrial-associated membranes (ER–MAMs), where it interacts with the endoplasmic reticulum Ca^2+^ ATPase (SERCA) pump to promote calcium overload in the mitochondria. This causes the loss of membrane potential and activation of the mitochondrial apoptotic process through the release of cytochrome c [21]. We retraced such pathways in thyroid cancer cells showing that it is induced by KRX-C7 treatment both in vitro and in vivo. Indeed, KRX-C7 significantly increases p53 levels and its interaction with SERCA in the ER. Moreover, it induces a calcium overload in mitochondria which results in the release of cytochrome c from mitochondria into the cytosol. Altogether these findings are of great interest in the cancer field for the identification of a novel strategy to reduce thyroid cancer progression mediated by the inhibition of GRK2 activity. Several kinase inhibitors have been proposed in the past decades to treat pathological conditions which were dependent on excessive GRK2 activity, such as cardiac damage [35]. In this context, we designed a novel strategy of inhibition of GRK2 by mimicking the HJ loop of the kinase which was effective for the treatment of cardiac hypertrophy [36] and of diabetes and its cardiovascular complications [34]. Diabetes has also been associated with a high risk of developing thyroid cancer [37]; thus, our inhibitor could be effective in the treatment of comorbidities associated with metabolic diseases (cardiovascular diseases and cancer). Further experiments will be needed to prove such a hypothesis; however, our data propose KRX-C7 as a promising treatment for undifferentiated thyroid cancer.

## 5. Conclusions

Our data demonstrated for the first time a close interaction between GRK2 and p53 in cancer which is responsible for kinase-dependent tumor growth. Moreover, we identify the inhibition of GRK2 activity as a potential strategy to delay tumor progression. These data strongly contribute to increasing the knowledge in tumor biology and suggest a novel molecule to use for therapeutic purposes.

## Supplementary Materials

The following are available online at https://www.mdpi.com/2072-6694/12/12/3530/s1: Figure S1: SF 1: KRX-C7 inhibits KAT-4 cell proliferation.
