# Supplementary material for: Exploiting GRK2 Inhibition as a Therapeutic Option in Experimental Cancer Treatment: Role of p53-Induced Mitochondrial Apoptosis
Source: Cancers (Basel). 2020 Nov 26;12(12):3530. doi: 10.3390/cancers12123530 (PMC7760517; doi:10.3390/cancers12123530)
Supplement: Supplementary file 1 [file cancers-12-03530-s001.zip › cancers-1016979-Supplementary Materials/cancers-1016979-Supplementary Figure 1.docx]

Supplementary Materials: Exploiting GRK2 Inhibition as a Therapeutic Option in Experimental Cancer Treatment: Role of p53-Induced Mitochondrial Apoptosis

Jessica Gambardella, AntonellaFiordelisi, Gaetano Santulli, Michele Ciccarelli, Federica Andrea Cerasuolo, Marina Sala, Eduardo Sommella, Pietro Campiglia, Maddalena Illario, Guido Iaccarino, and Daniela Sorriento


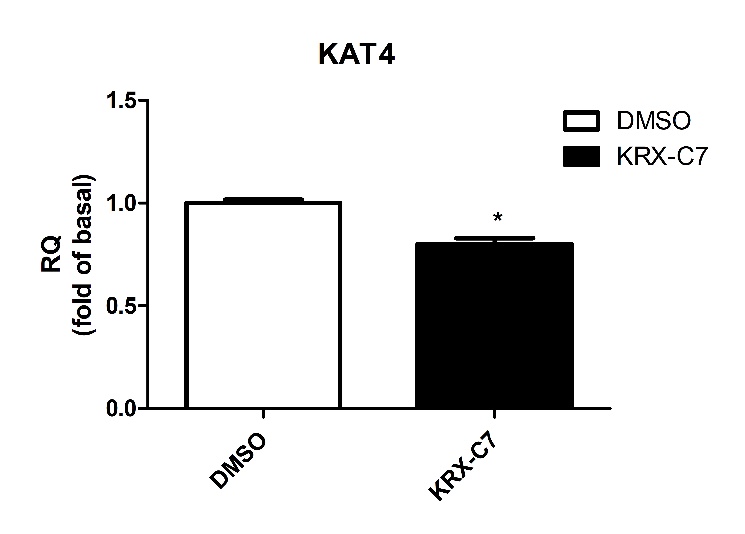


**Figure 1.** SF 1: KRX-C7 inhibits KAT-4 cell proliferation. KAT-4 cells were treated with DMSO or KRX-C7 and cell proliferation was evaluated. KRX-C7 inhibited KAT-4 proliferation; * *p* < 0.05 vs DMSO.

**Publisher's Note:** MDPI stays neutral with regard to jurisdictional claims in published maps and institutional affiliations.


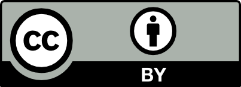
© 2020 by the authors. Licensee MDPI, Basel, Switzerland. This article is an open access article distributed under the terms and conditions of the Creative Commons Attribution (CC BY) license (http://creativecommons.org/licenses/by/4.0/).
