# Supplementary figures and images for: Exploiting GRK2 Inhibition as a Therapeutic Option in Experimental Cancer Treatment: Role of p53-Induced Mitochondrial Apoptosis
Source: Cancers (Basel). 2020 Nov 26;12(12):3530. doi: 10.3390/cancers12123530 (PMC7760517; doi:10.3390/cancers12123530)

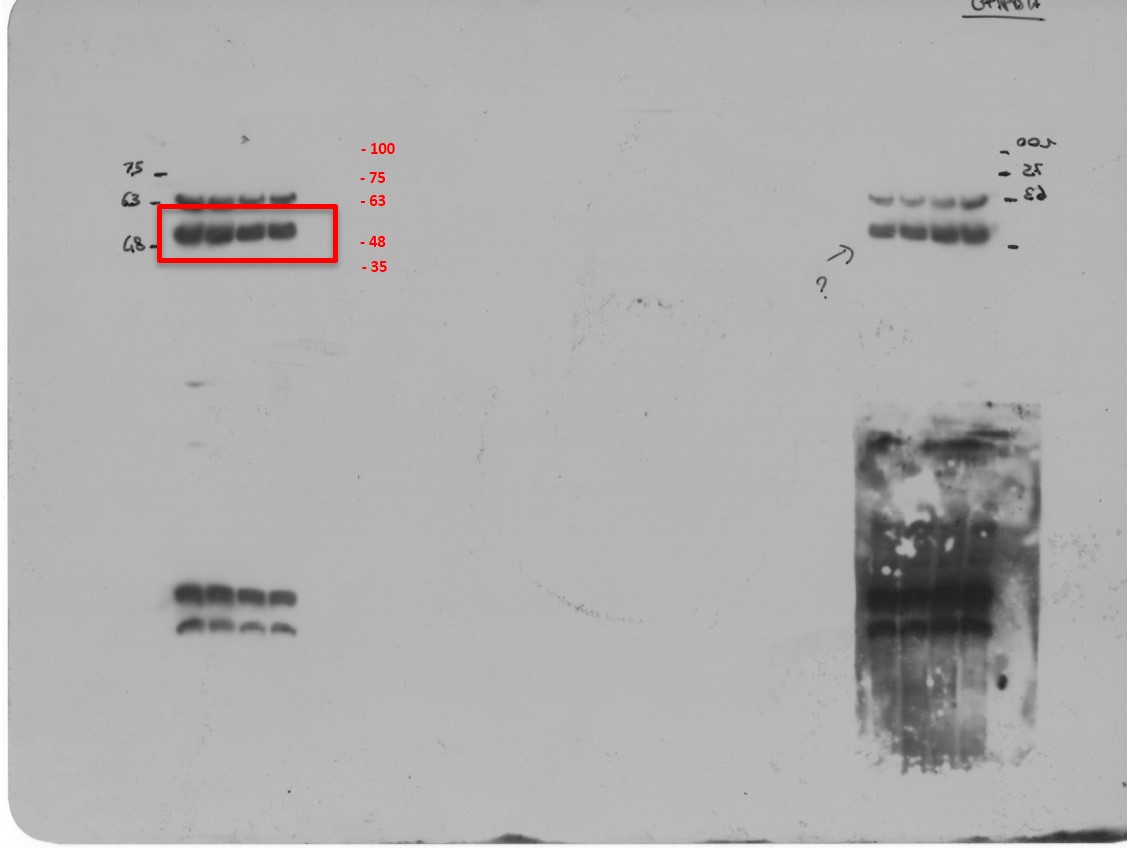

Supplement: Supplementary file 1 [file cancers-12-03530-s001.zip › cancers-1016979-Supplementary Materials/BLOTS/FIGURE 1/PANEL A/ACTIN 1.jpg]

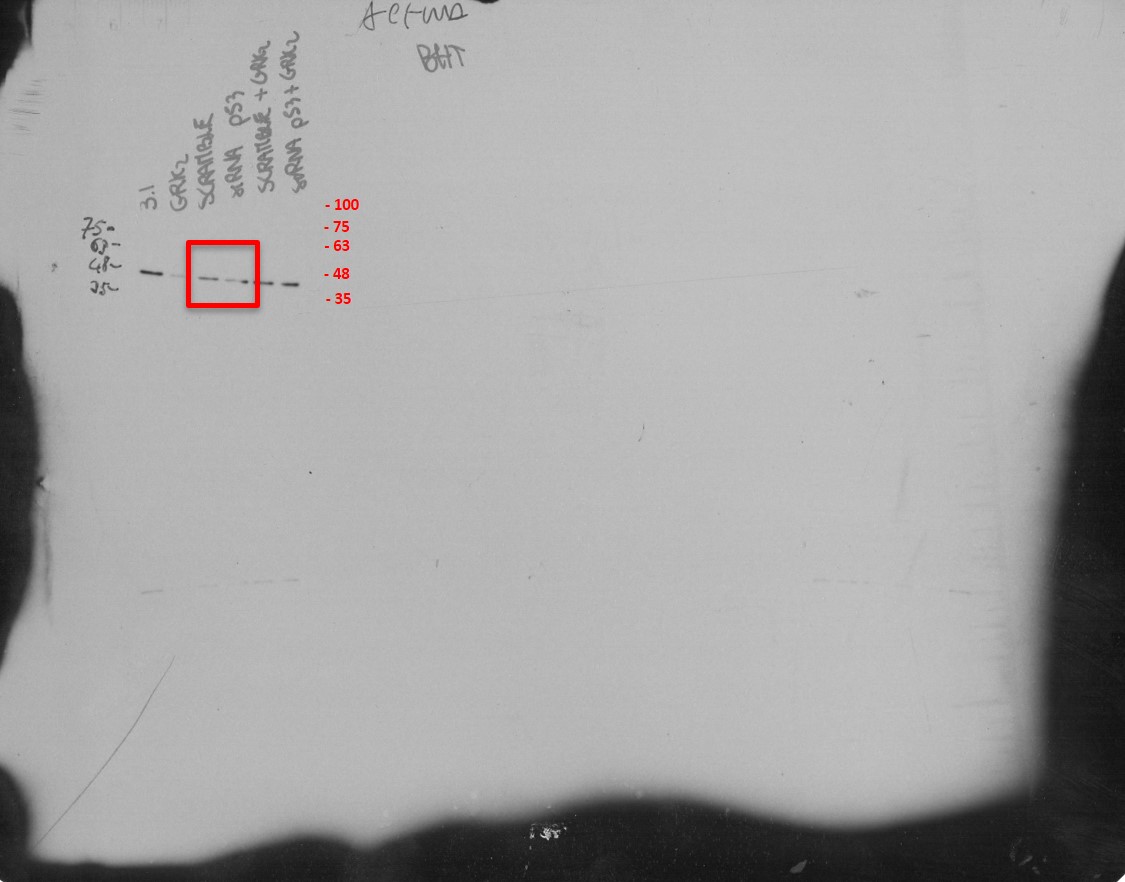

Supplement: Supplementary file 1 [file cancers-12-03530-s001.zip › cancers-1016979-Supplementary Materials/BLOTS/FIGURE 1/PANEL A/ACTIN 2.jpg]

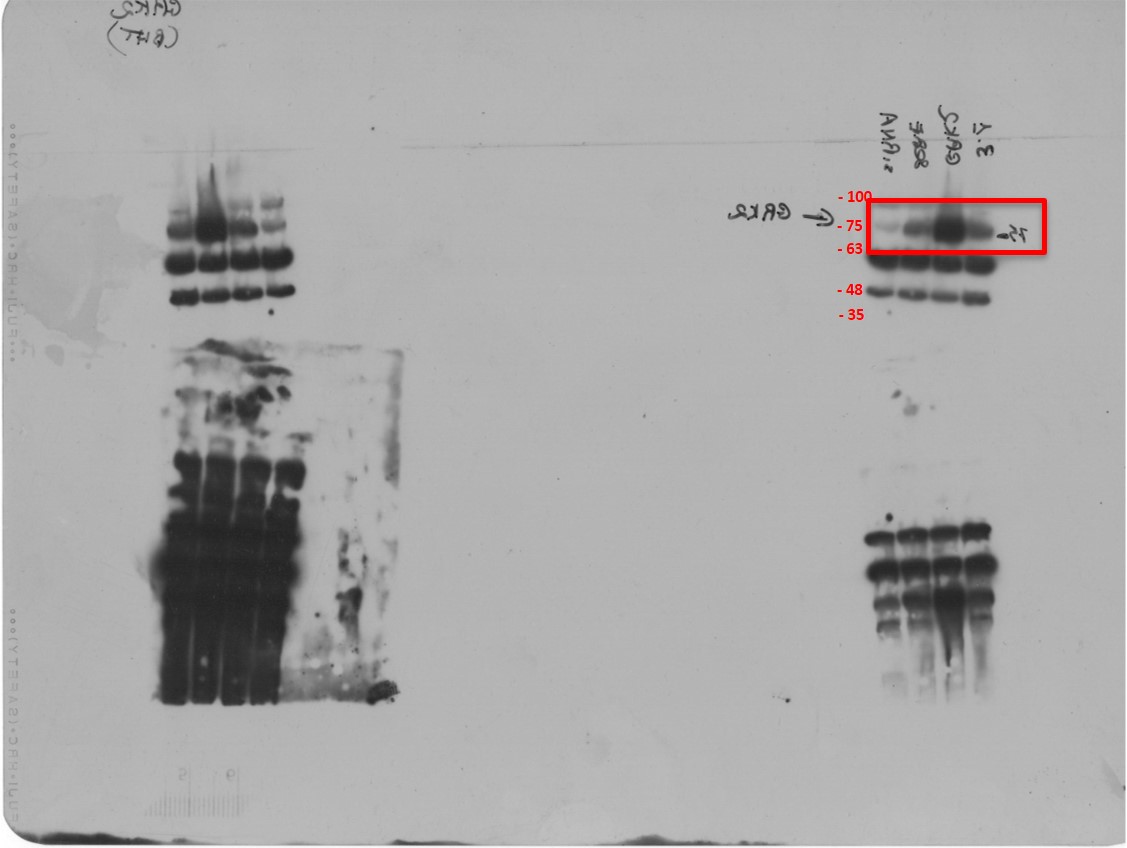

Supplement: Supplementary file 1 [file cancers-12-03530-s001.zip › cancers-1016979-Supplementary Materials/BLOTS/FIGURE 1/PANEL A/GRK2.jpg]

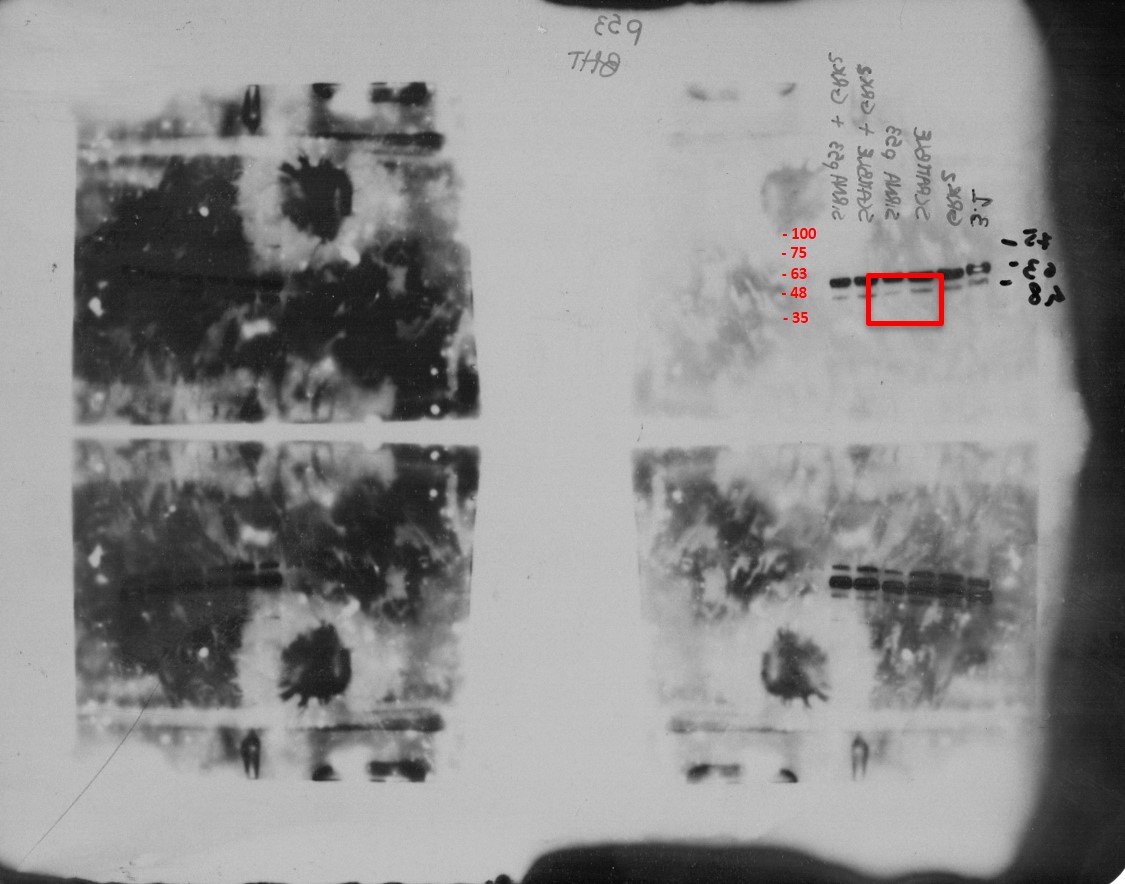

Supplement: Supplementary file 1 [file cancers-12-03530-s001.zip › cancers-1016979-Supplementary Materials/BLOTS/FIGURE 1/PANEL A/P53.jpg]

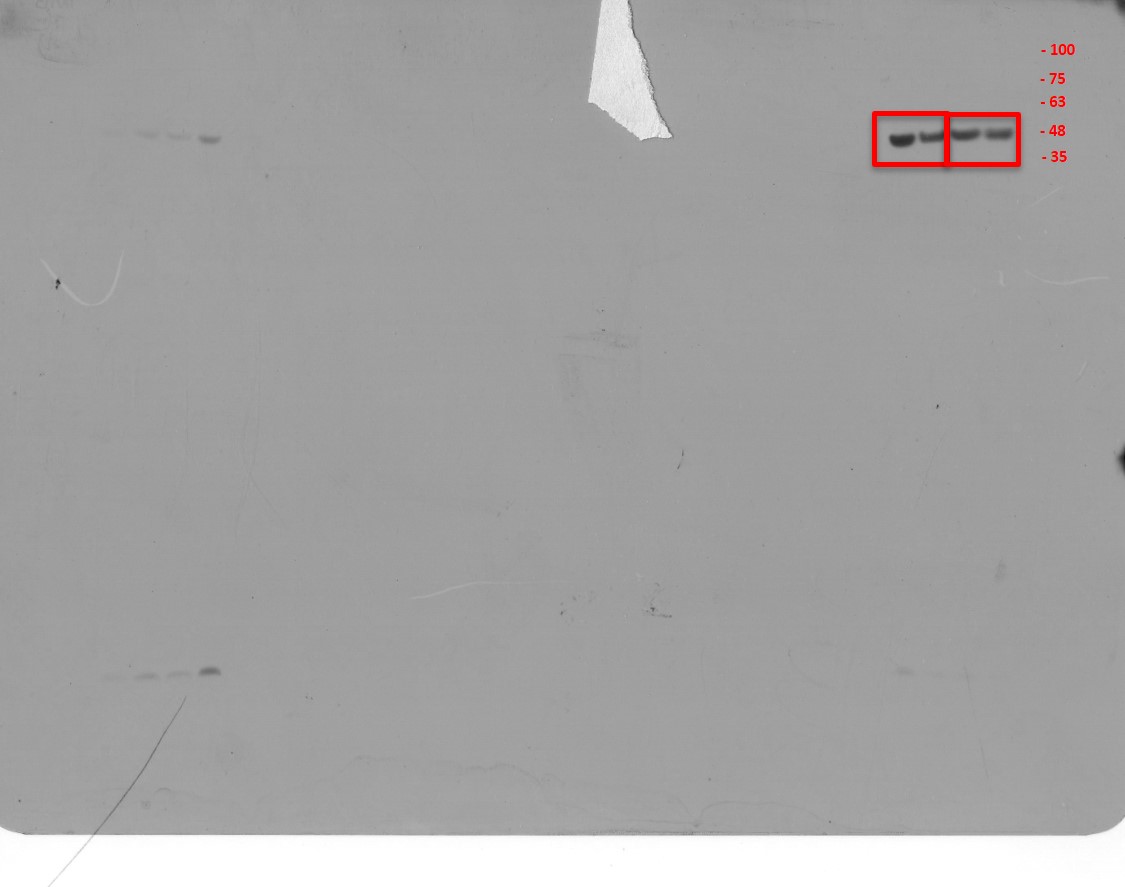

Supplement: Supplementary file 1 [file cancers-12-03530-s001.zip › cancers-1016979-Supplementary Materials/BLOTS/FIGURE 1/PANEL C/ACTIN BHT FRO.jpg]

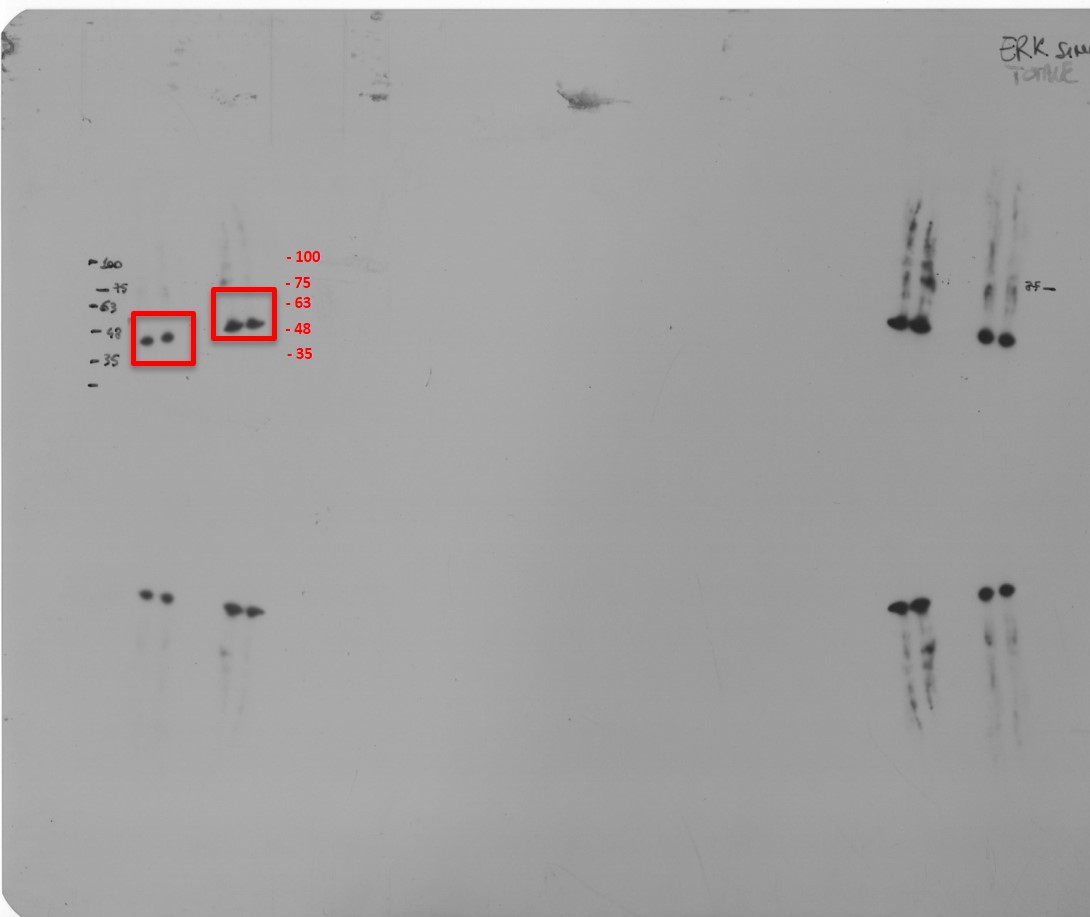

Supplement: Supplementary file 1 [file cancers-12-03530-s001.zip › cancers-1016979-Supplementary Materials/BLOTS/FIGURE 1/PANEL C/ERK2 BHT - FRO.jpg]

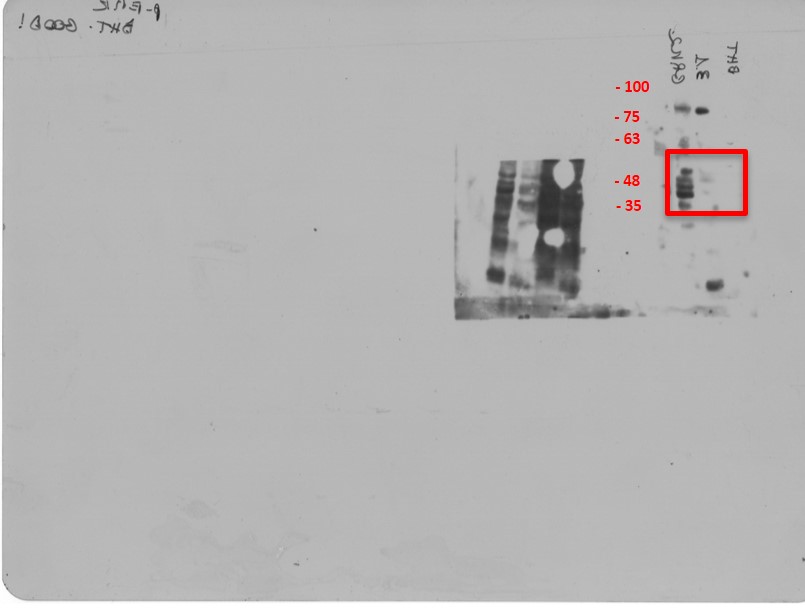

Supplement: Supplementary file 1 [file cancers-12-03530-s001.zip › cancers-1016979-Supplementary Materials/BLOTS/FIGURE 1/PANEL C/pERK BHT.jpg]

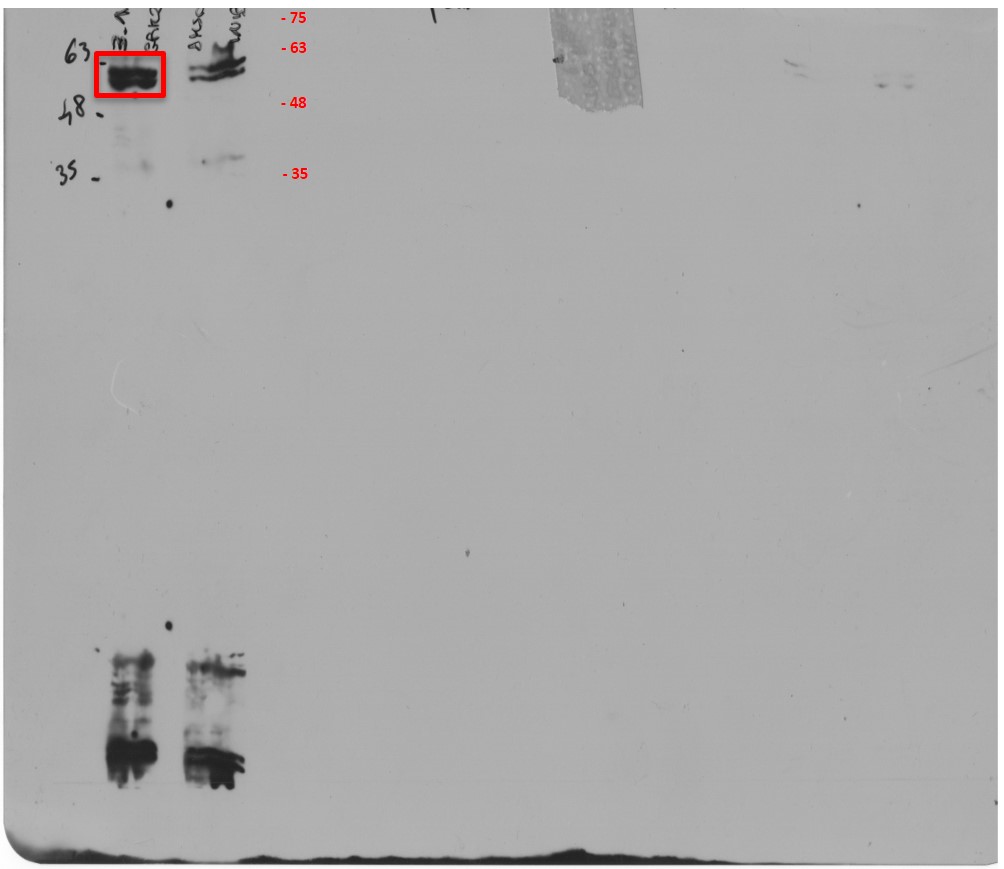

Supplement: Supplementary file 1 [file cancers-12-03530-s001.zip › cancers-1016979-Supplementary Materials/BLOTS/FIGURE 1/PANEL C/pERK FRO.jpg]

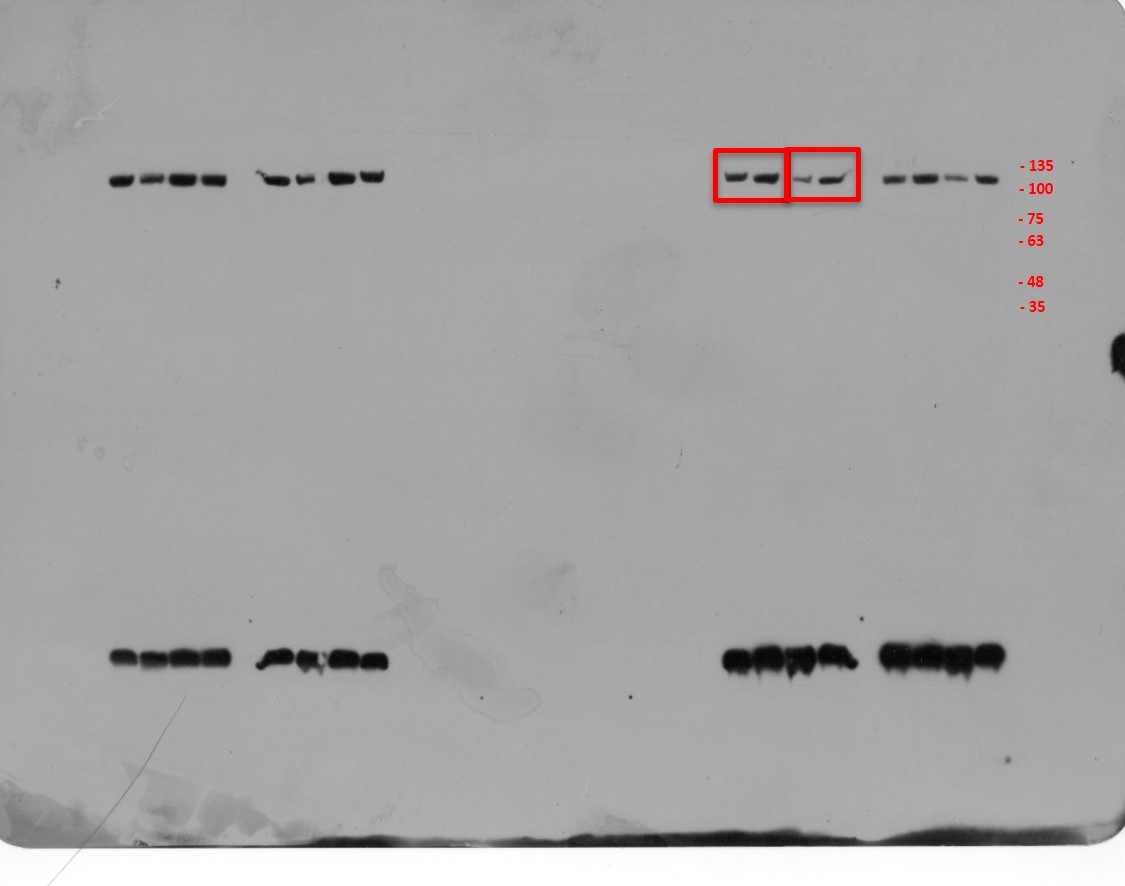

Supplement: Supplementary file 1 [file cancers-12-03530-s001.zip › cancers-1016979-Supplementary Materials/BLOTS/FIGURE 1/PANEL C/pRB BHT FRO.jpg]

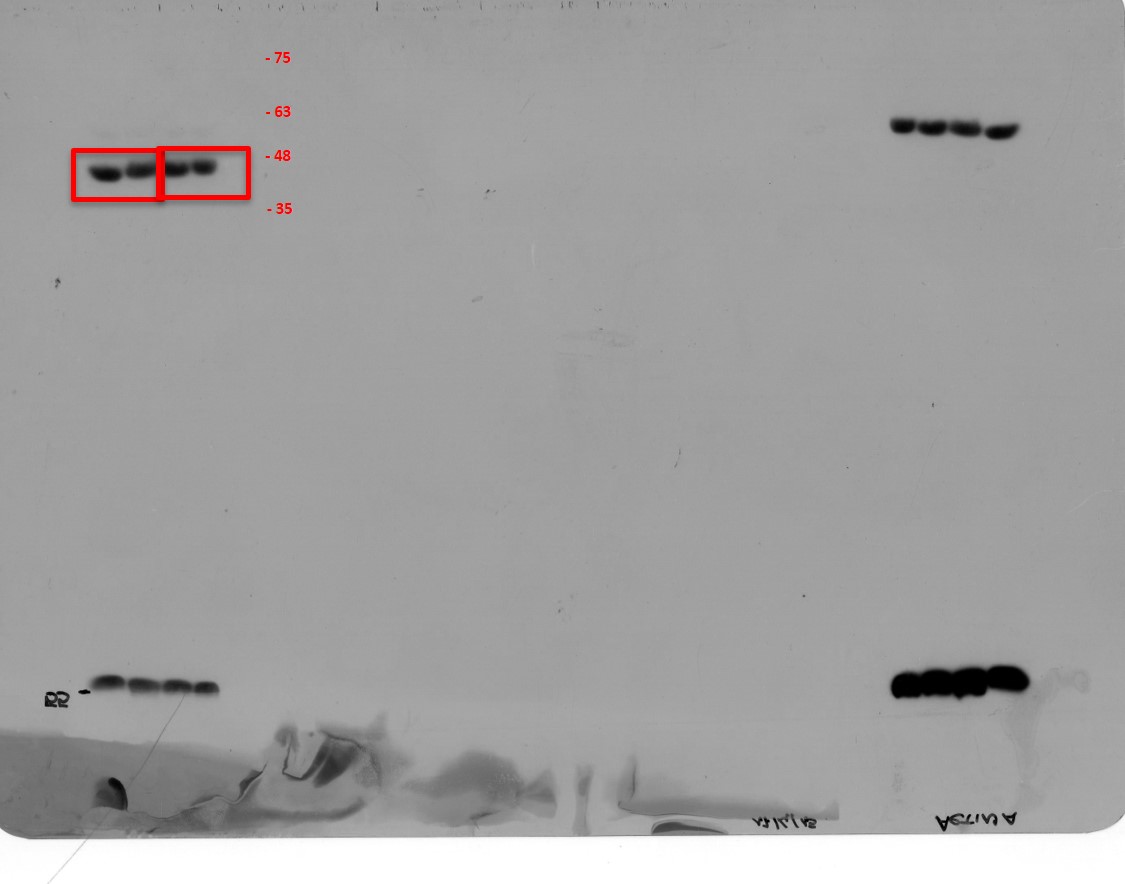

Supplement: Supplementary file 1 [file cancers-12-03530-s001.zip › cancers-1016979-Supplementary Materials/BLOTS/FIGURE 1/PANEL D/ACTINA FRO-BHT.jpg]

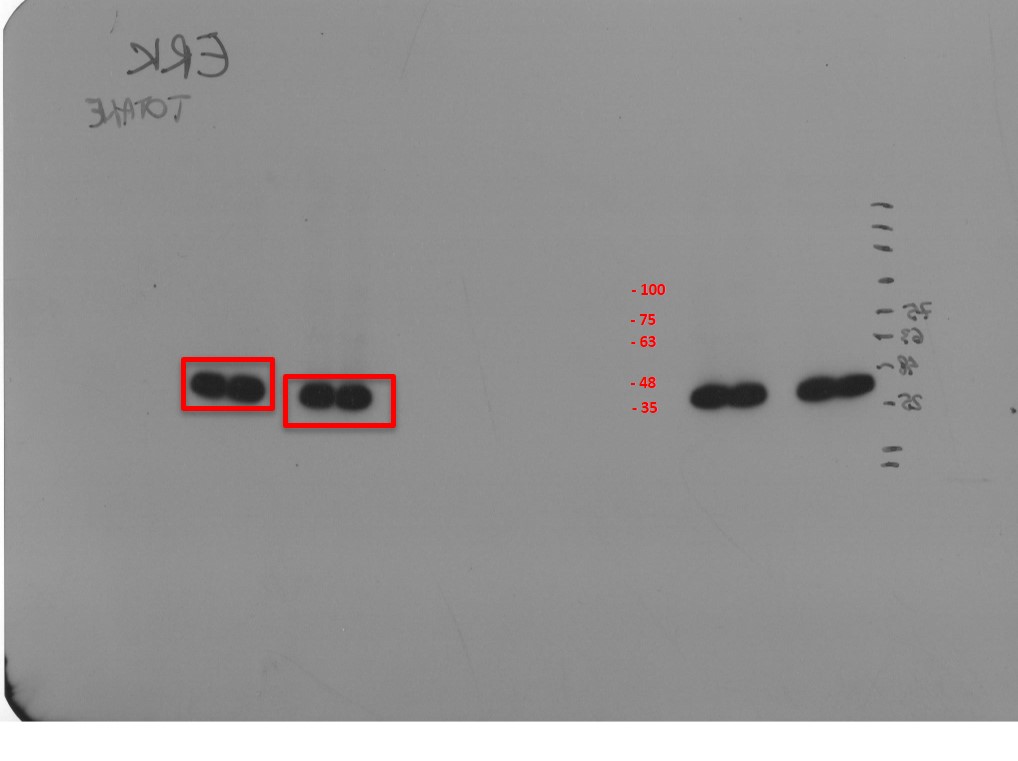

Supplement: Supplementary file 1 [file cancers-12-03530-s001.zip › cancers-1016979-Supplementary Materials/BLOTS/FIGURE 1/PANEL D/ERK BHT FRO.jpg]

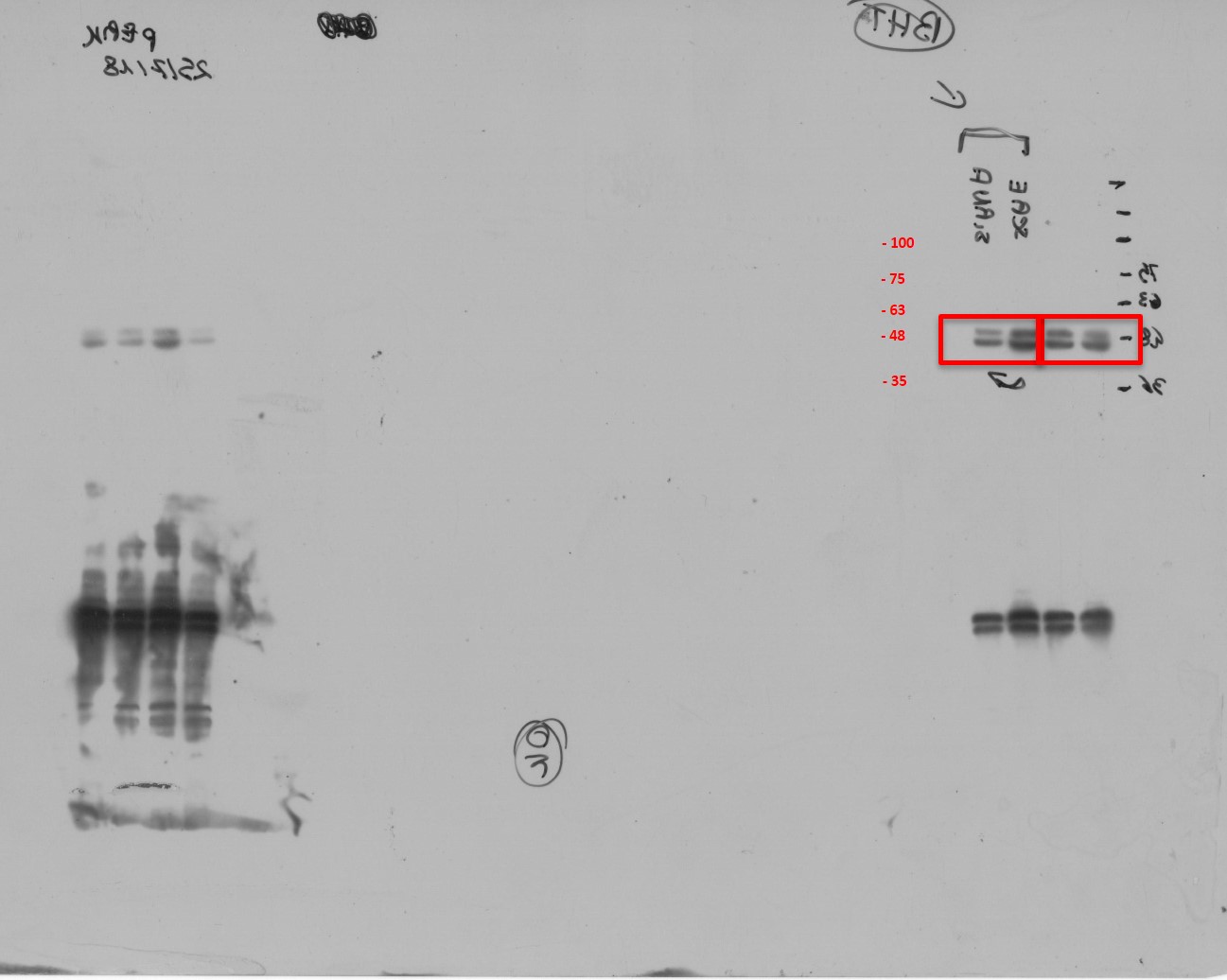

Supplement: Supplementary file 1 [file cancers-12-03530-s001.zip › cancers-1016979-Supplementary Materials/BLOTS/FIGURE 1/PANEL D/pERK BHT FRO.jpg]

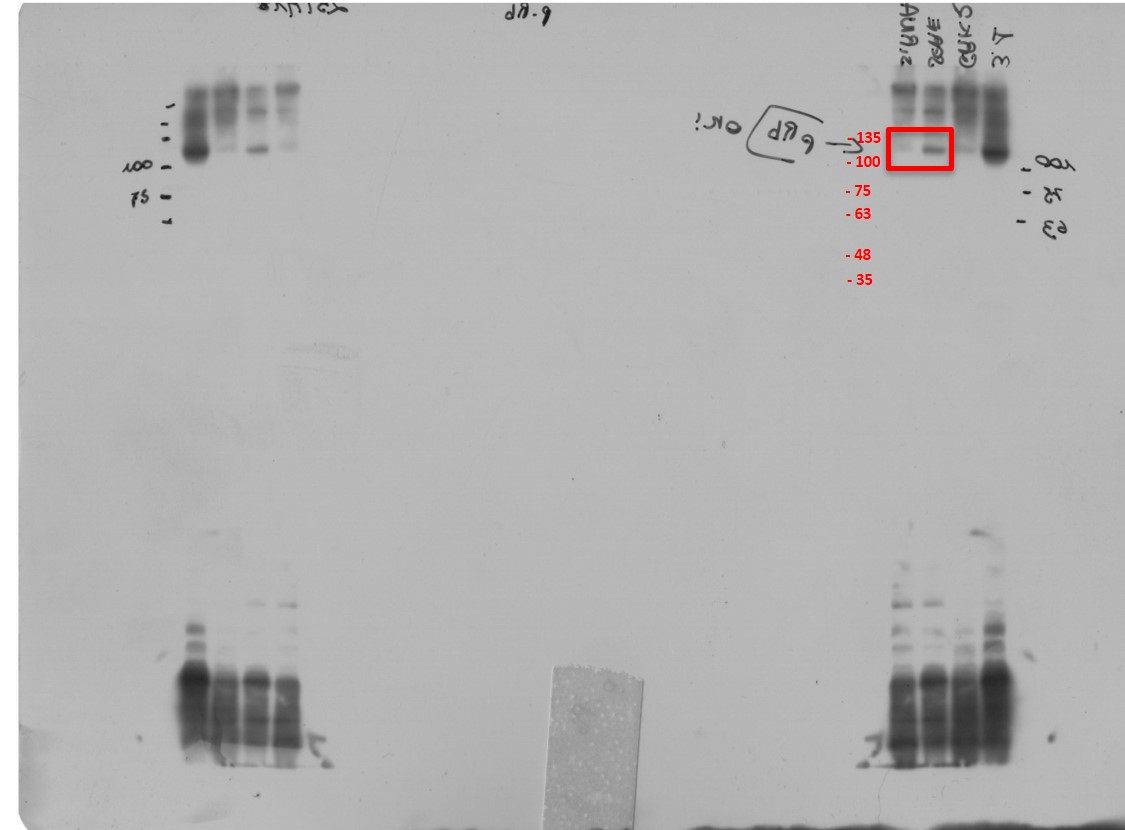

Supplement: Supplementary file 1 [file cancers-12-03530-s001.zip › cancers-1016979-Supplementary Materials/BLOTS/FIGURE 1/PANEL D/pRB BHT.jpg]

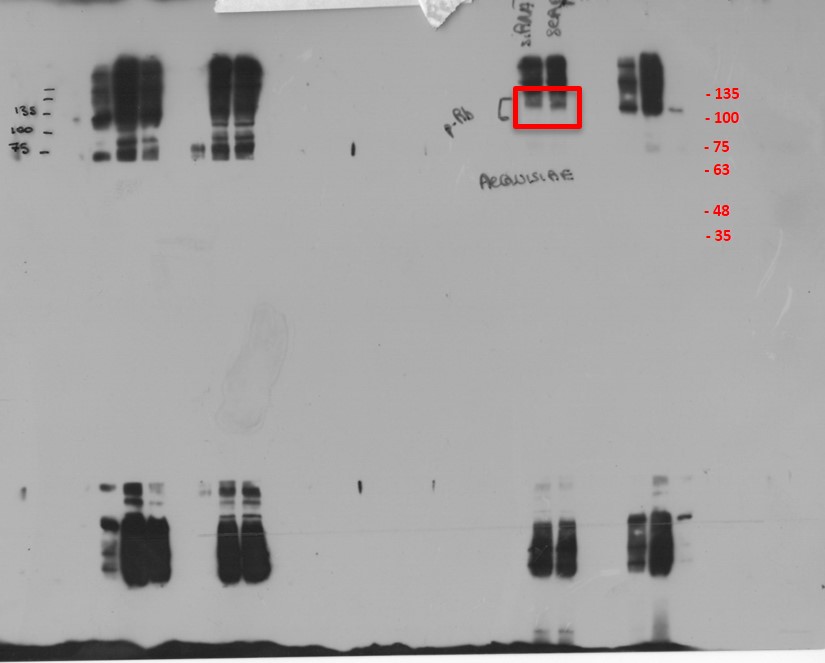

Supplement: Supplementary file 1 [file cancers-12-03530-s001.zip › cancers-1016979-Supplementary Materials/BLOTS/FIGURE 1/PANEL D/pRB FRO.jpg]

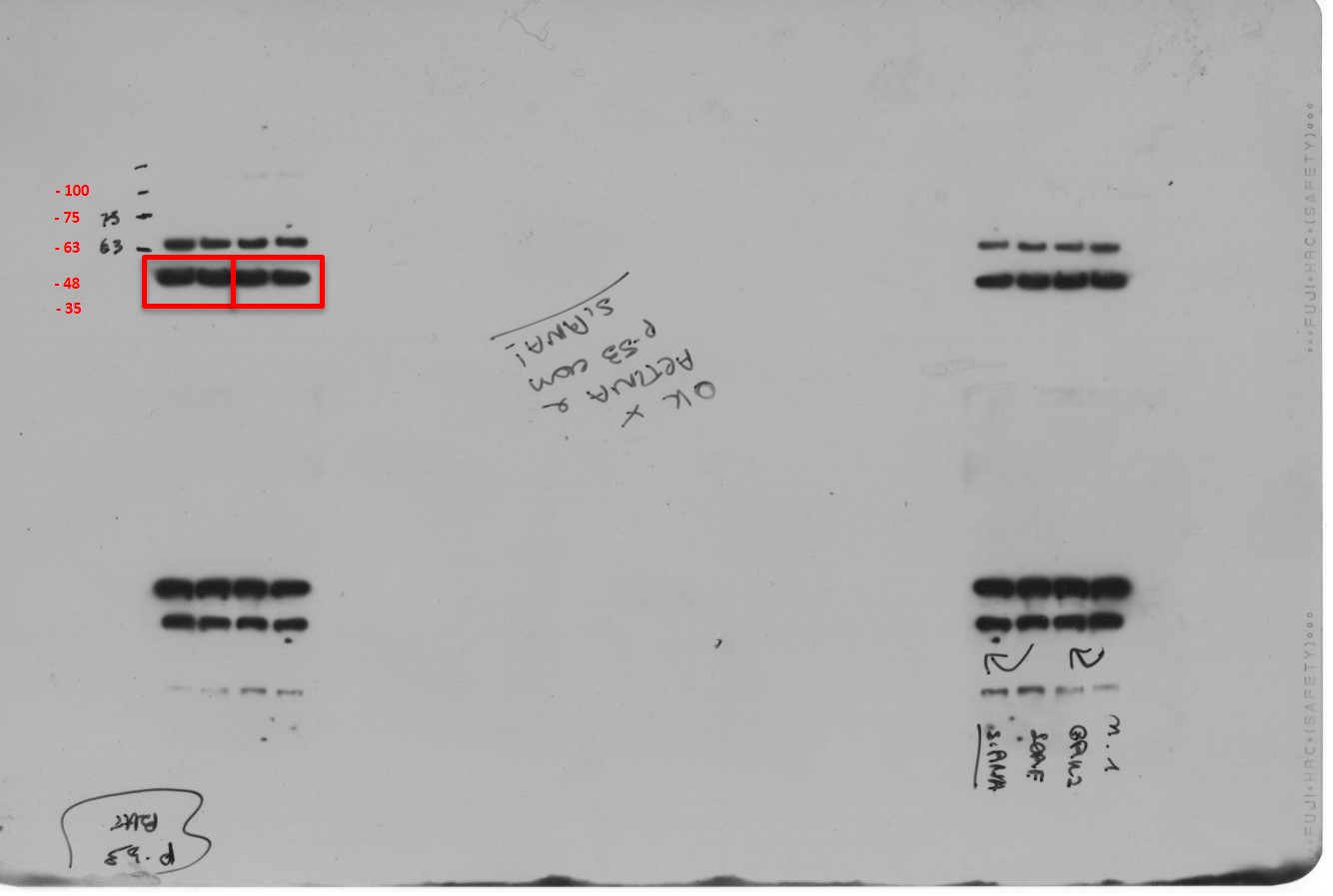

Supplement: Supplementary file 1 [file cancers-12-03530-s001.zip › cancers-1016979-Supplementary Materials/BLOTS/FIGURE 1/PANEL E/ACTIN.jpg]

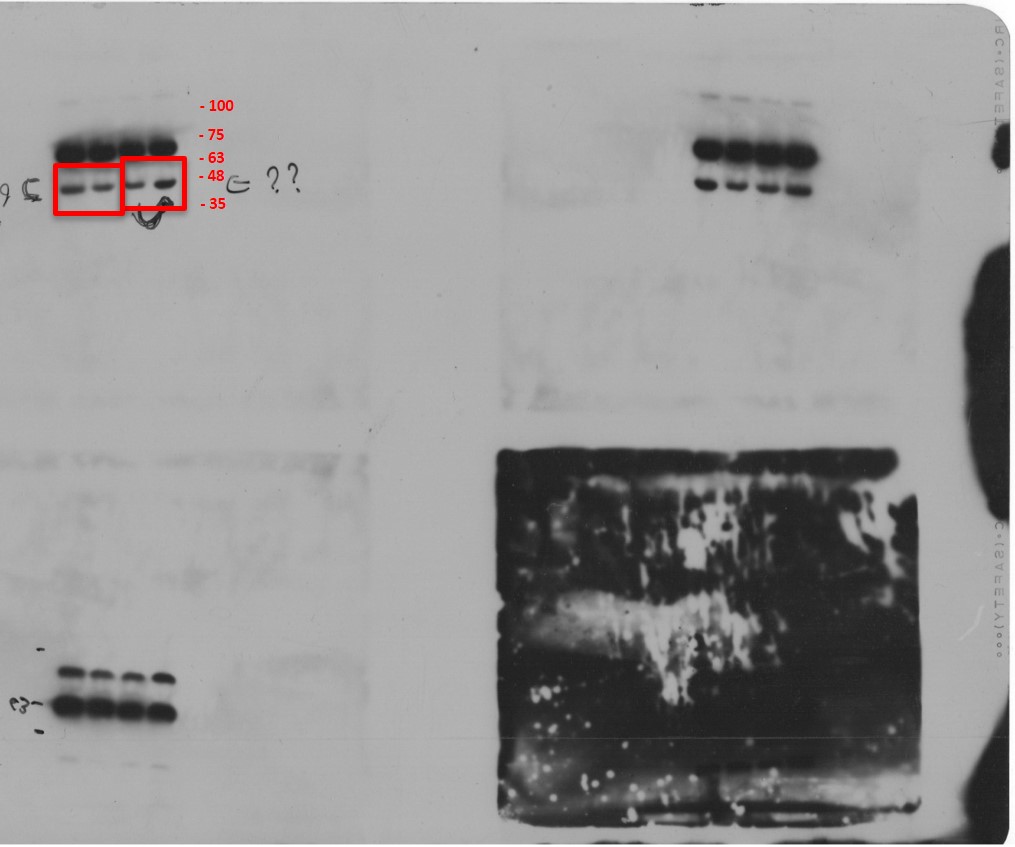

Supplement: Supplementary file 1 [file cancers-12-03530-s001.zip › cancers-1016979-Supplementary Materials/BLOTS/FIGURE 1/PANEL E/P53.jpg]

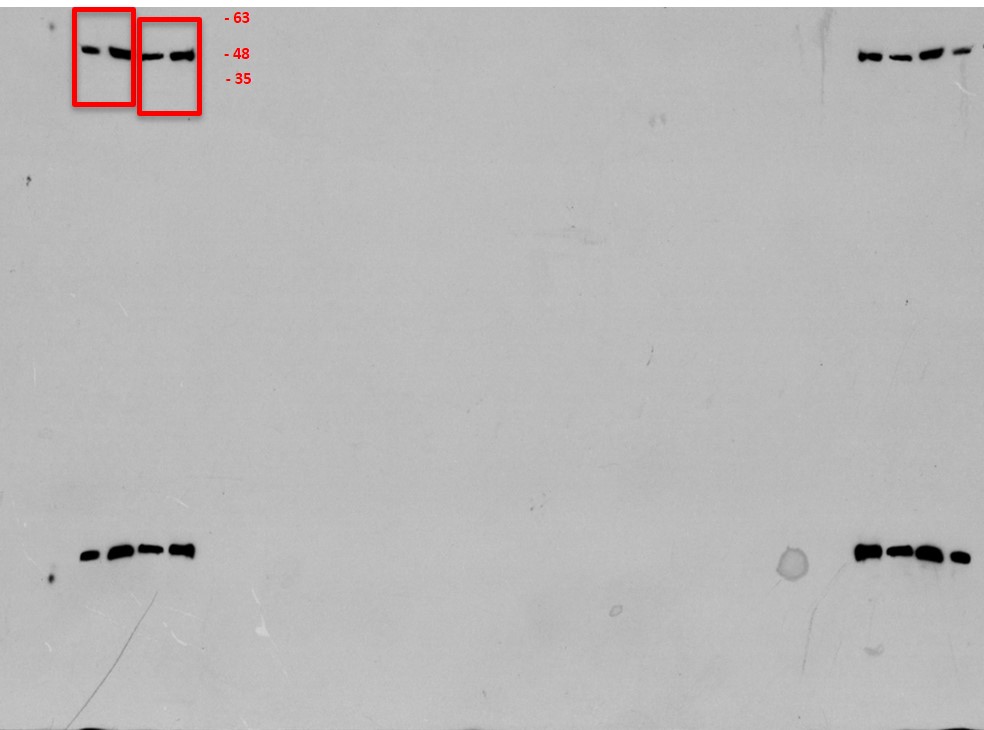

Supplement: Supplementary file 1 [file cancers-12-03530-s001.zip › cancers-1016979-Supplementary Materials/BLOTS/FIGURE 2/PANEL C/ACTIN BHT and FRO.jpg]

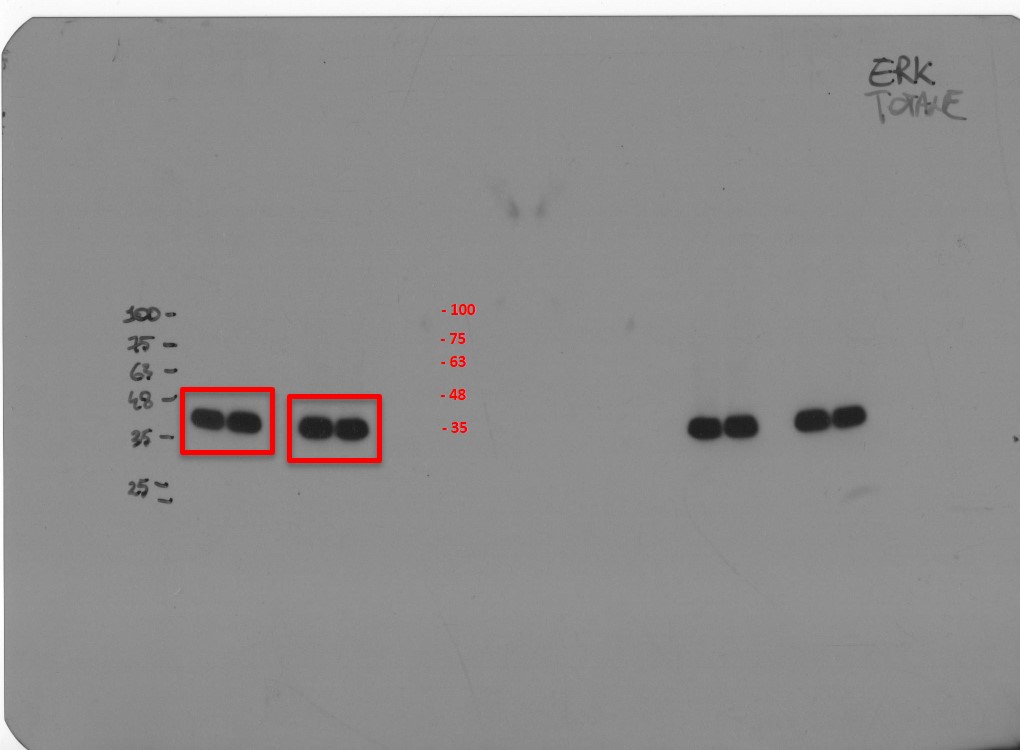

Supplement: Supplementary file 1 [file cancers-12-03530-s001.zip › cancers-1016979-Supplementary Materials/BLOTS/FIGURE 2/PANEL C/ERK TOT.jpg]

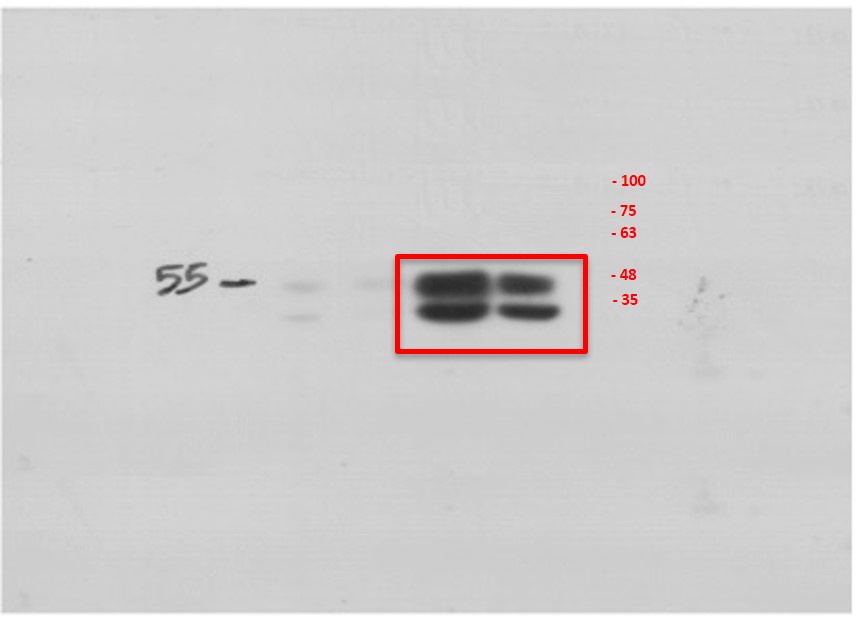

Supplement: Supplementary file 1 [file cancers-12-03530-s001.zip › cancers-1016979-Supplementary Materials/BLOTS/FIGURE 2/PANEL C/PERK BHT.jpg]

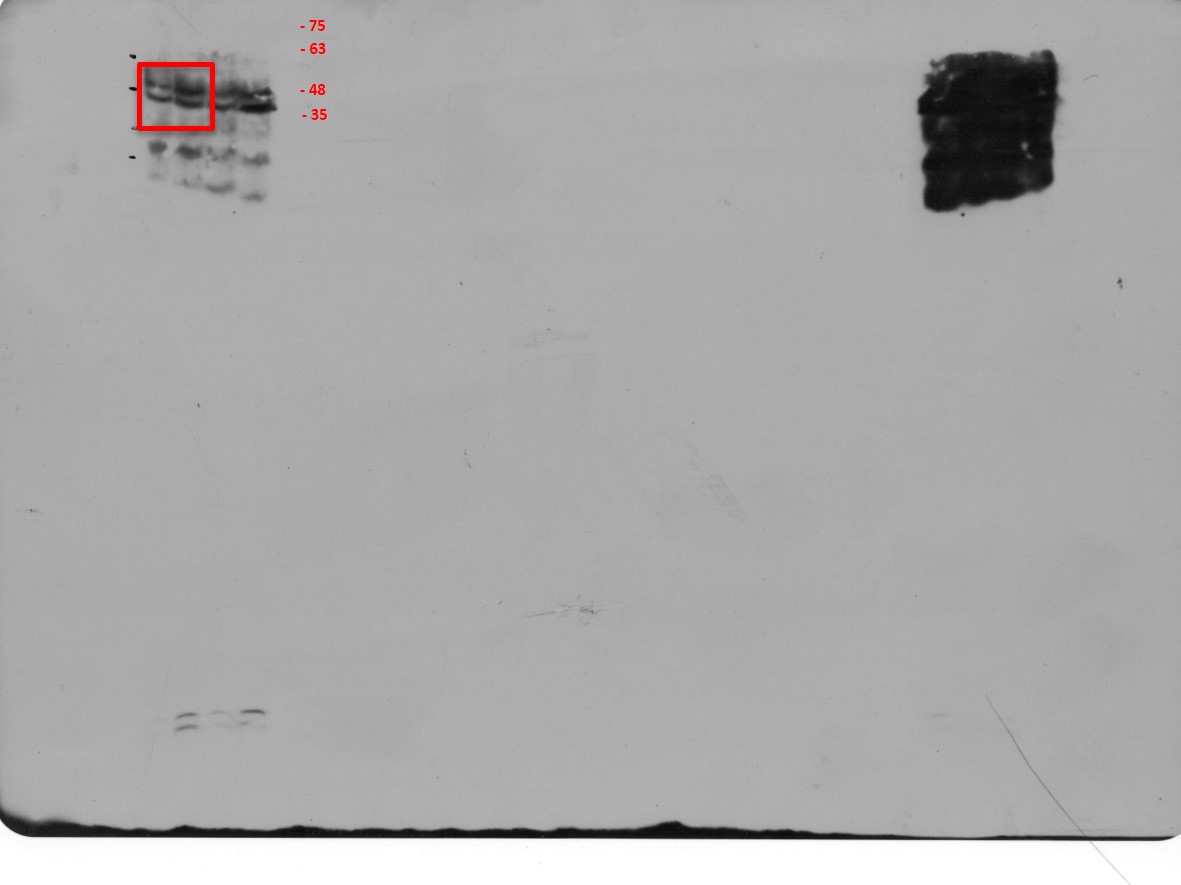

Supplement: Supplementary file 1 [file cancers-12-03530-s001.zip › cancers-1016979-Supplementary Materials/BLOTS/FIGURE 2/PANEL C/PERK FRO.jpg]

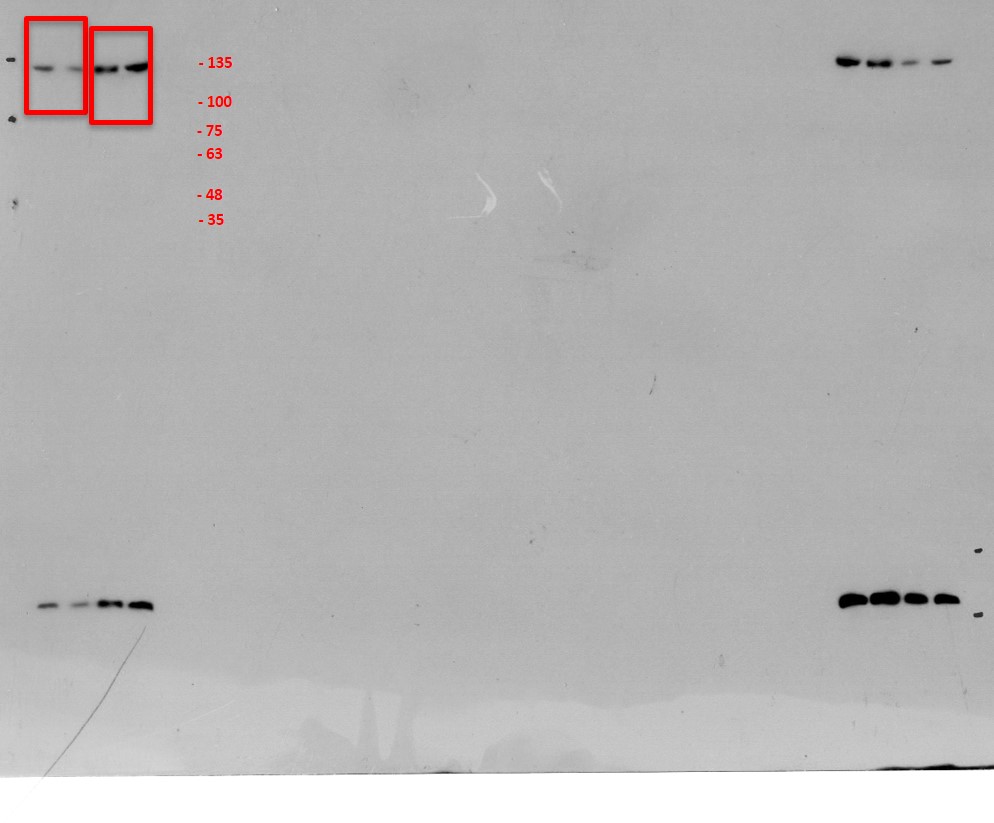

Supplement: Supplementary file 1 [file cancers-12-03530-s001.zip › cancers-1016979-Supplementary Materials/BLOTS/FIGURE 2/PANEL C/PRB BHT and FRO.jpg]

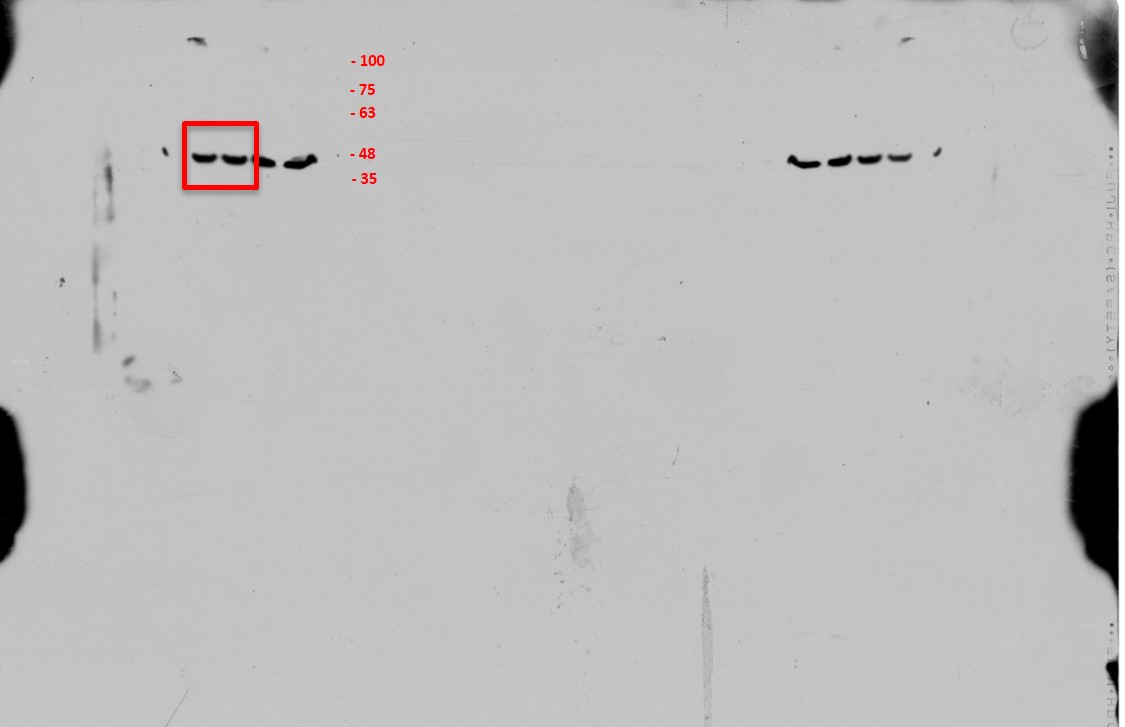

Supplement: Supplementary file 1 [file cancers-12-03530-s001.zip › cancers-1016979-Supplementary Materials/BLOTS/FIGURE 2/PANEL D/ACTIN PANEL D.jpg]

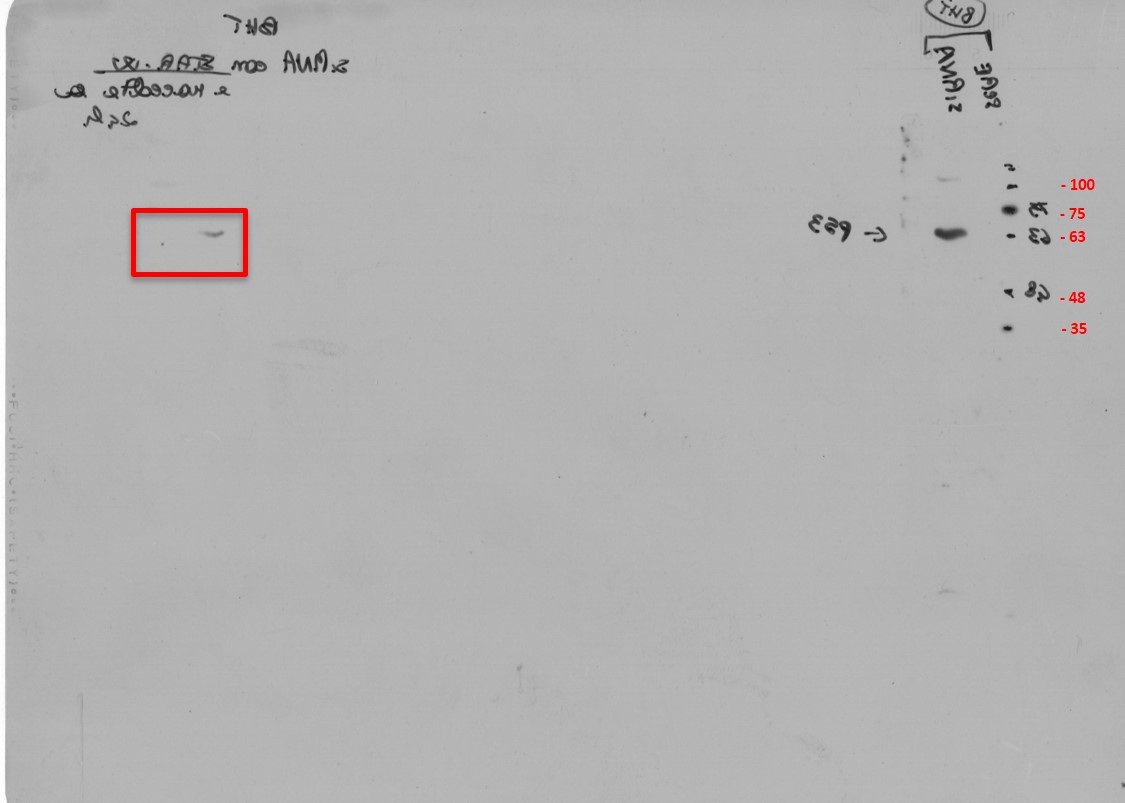

Supplement: Supplementary file 1 [file cancers-12-03530-s001.zip › cancers-1016979-Supplementary Materials/BLOTS/FIGURE 2/PANEL D/P53 PANEL D.jpg]

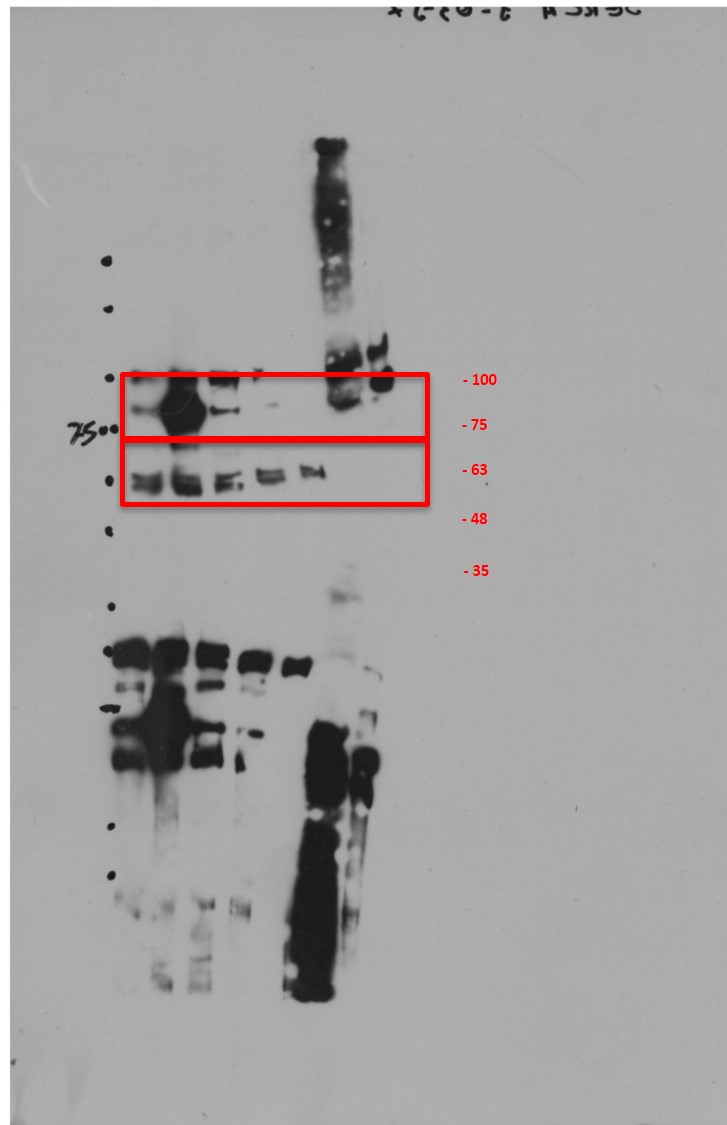

Supplement: Supplementary file 1 [file cancers-12-03530-s001.zip › cancers-1016979-Supplementary Materials/BLOTS/FIGURE 3/PANEL A/IMMUNOPRECIPITATE.jpg]

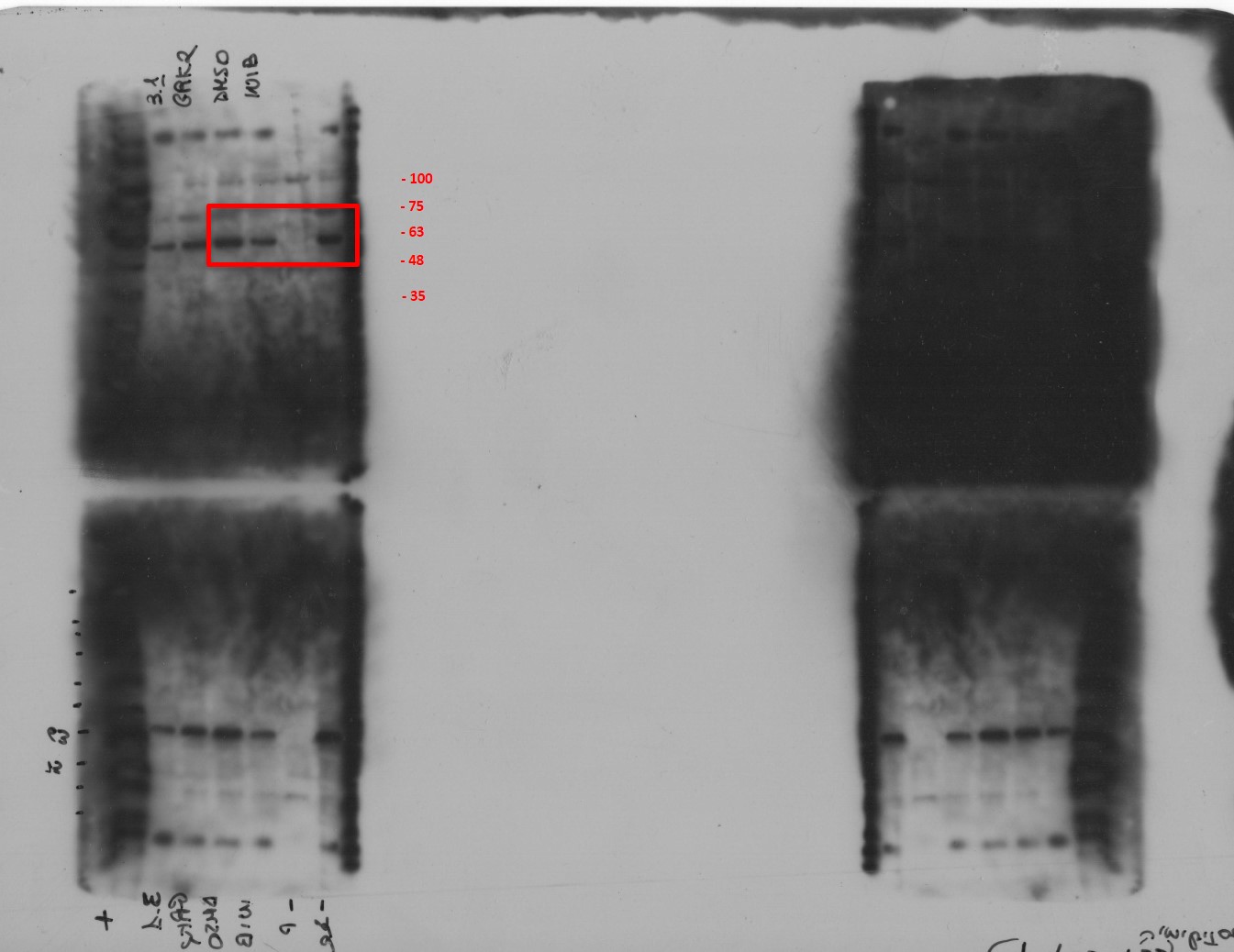

Supplement: Supplementary file 1 [file cancers-12-03530-s001.zip › cancers-1016979-Supplementary Materials/BLOTS/FIGURE 3/PANEL C/p-P53.jpg]

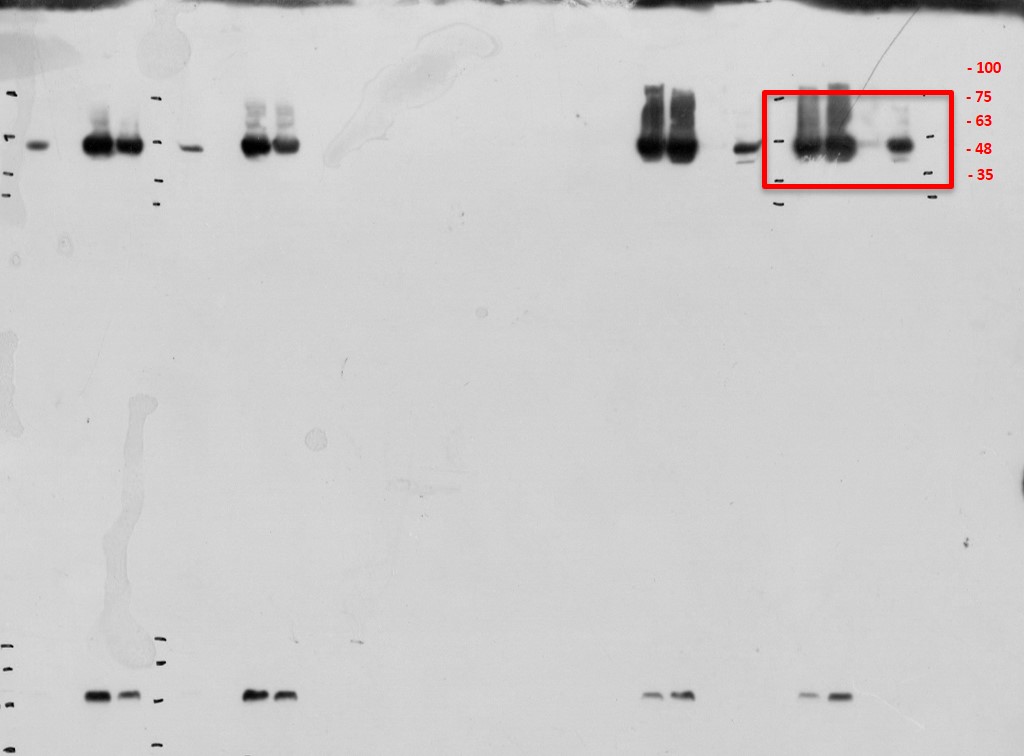

Supplement: Supplementary file 1 [file cancers-12-03530-s001.zip › cancers-1016979-Supplementary Materials/BLOTS/FIGURE 3/PANEL C/P53.jpg]

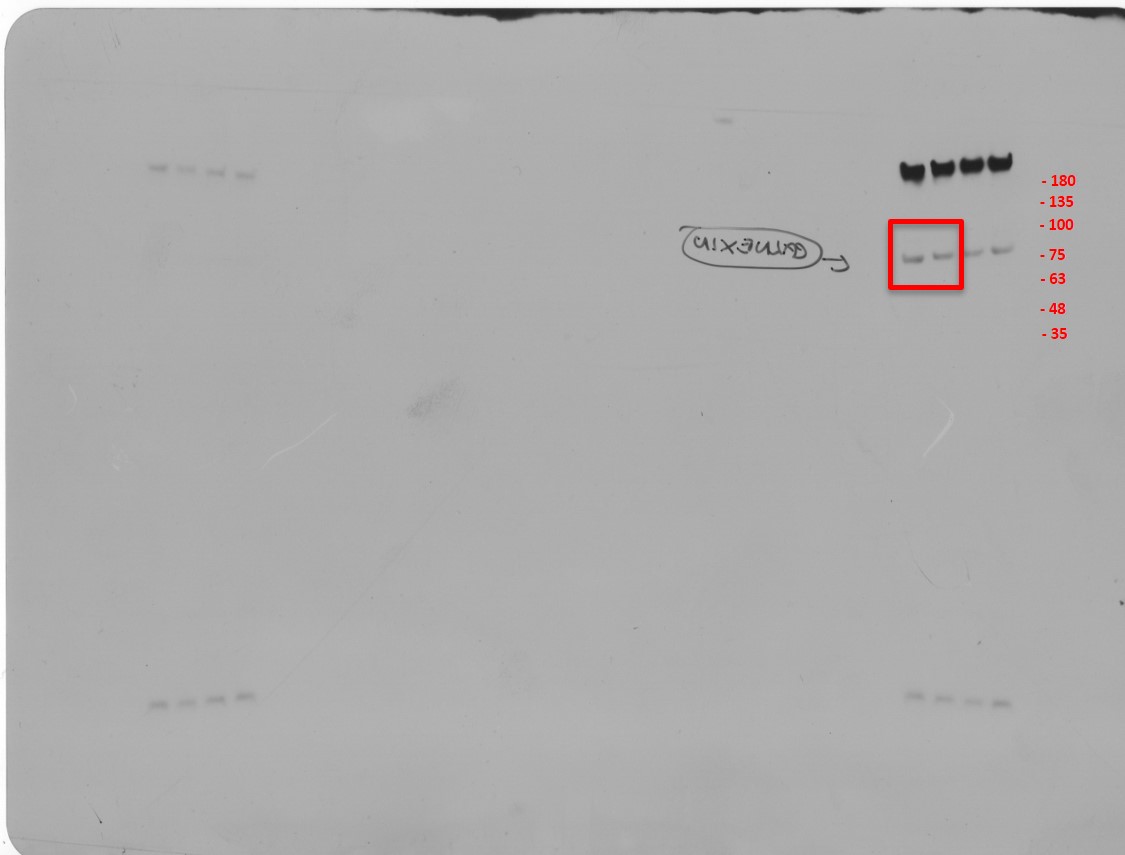

Supplement: Supplementary file 1 [file cancers-12-03530-s001.zip › cancers-1016979-Supplementary Materials/BLOTS/FIGURE 4/PANEL A/CALNEXIN.jpg]

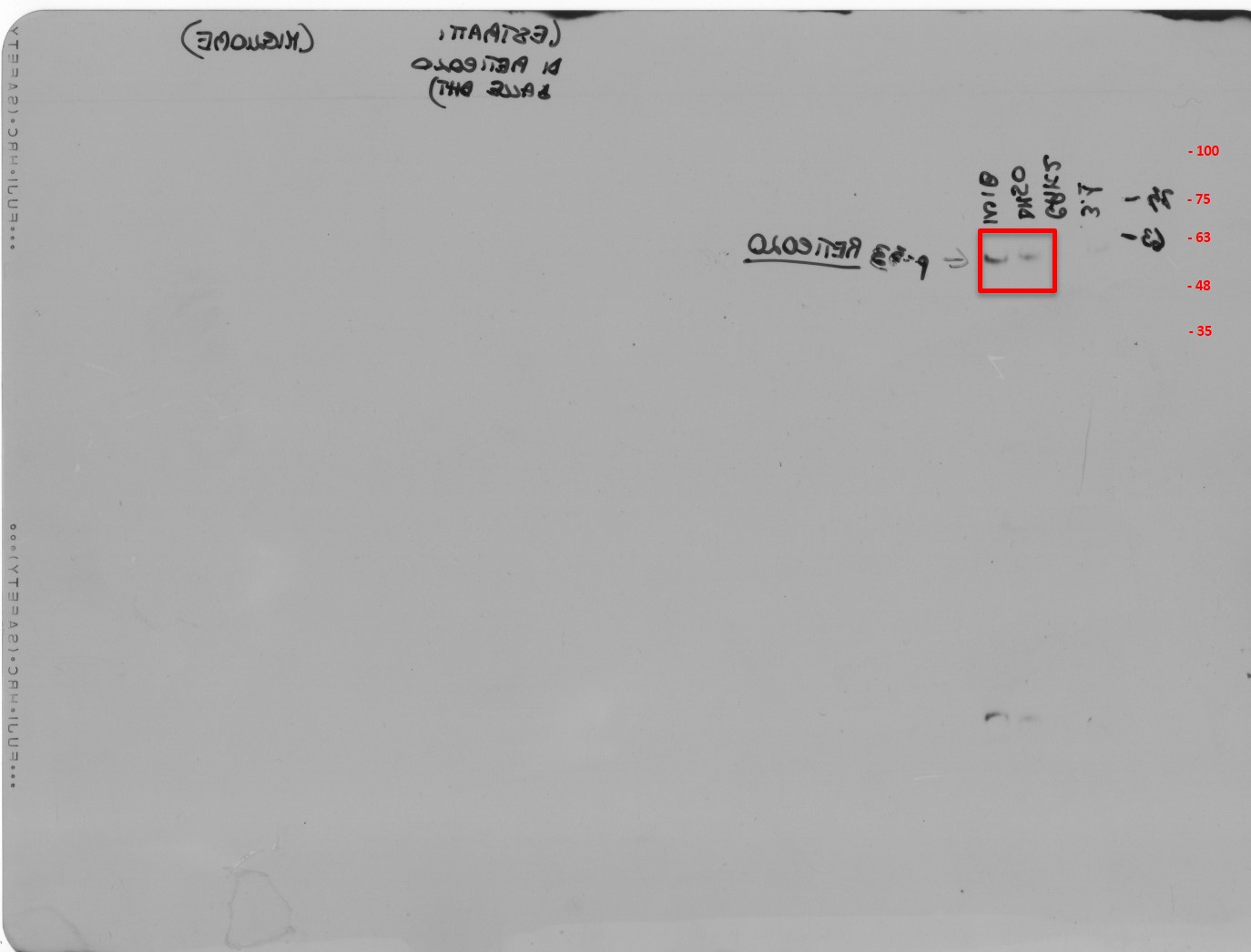

Supplement: Supplementary file 1 [file cancers-12-03530-s001.zip › cancers-1016979-Supplementary Materials/BLOTS/FIGURE 4/PANEL A/P53.jpg]

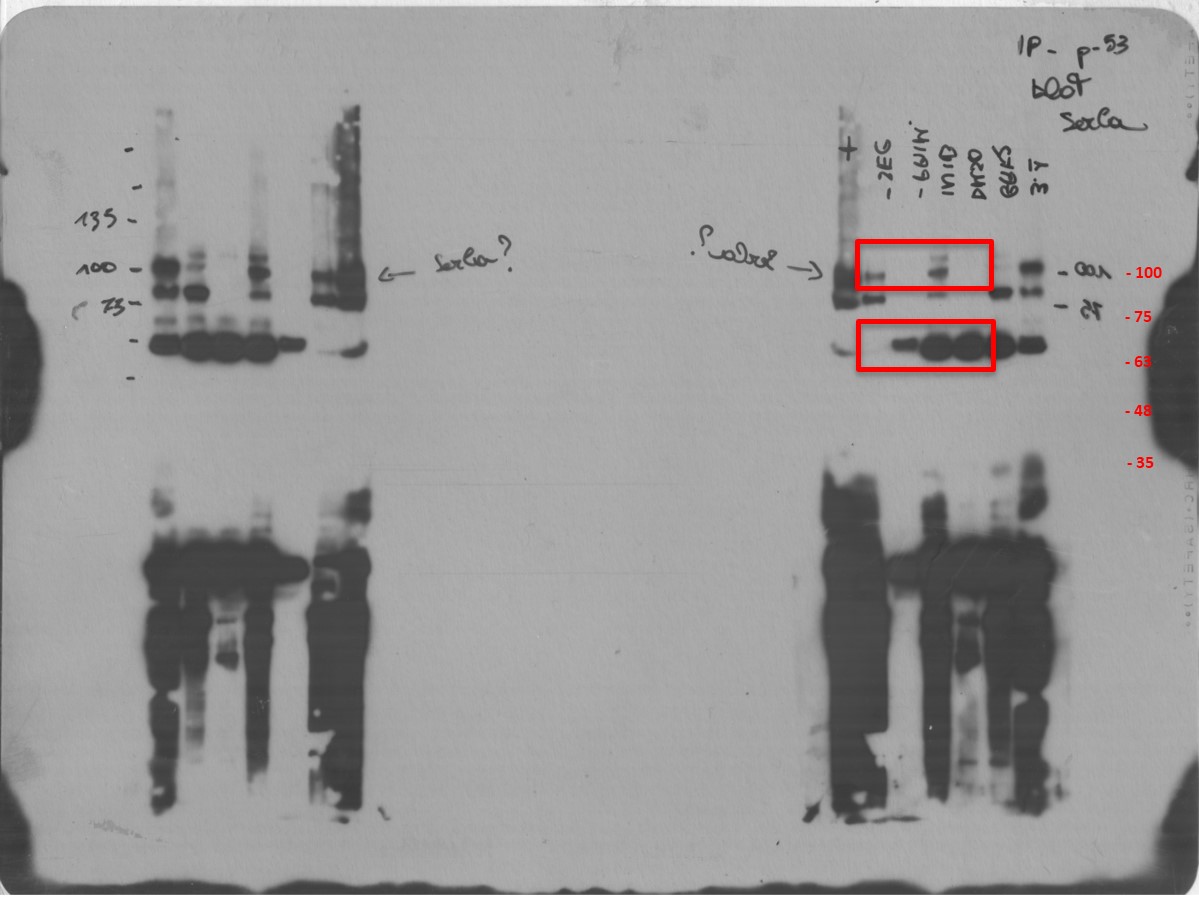

Supplement: Supplementary file 1 [file cancers-12-03530-s001.zip › cancers-1016979-Supplementary Materials/BLOTS/FIGURE 4/PANEL B/SERCA.jpg]

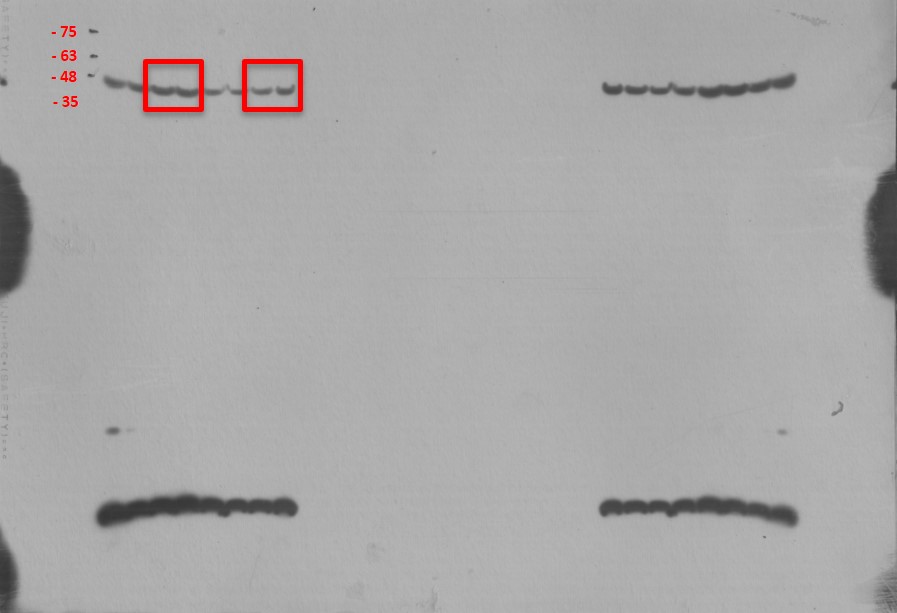

Supplement: Supplementary file 1 [file cancers-12-03530-s001.zip › cancers-1016979-Supplementary Materials/BLOTS/FIGURE 4/PANEL D/ACTIN.jpg]

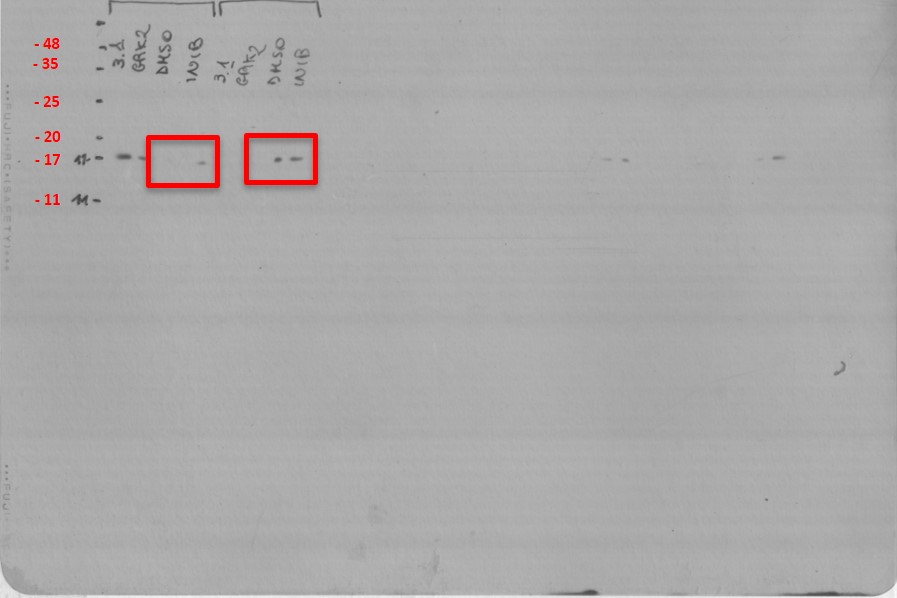

Supplement: Supplementary file 1 [file cancers-12-03530-s001.zip › cancers-1016979-Supplementary Materials/BLOTS/FIGURE 4/PANEL D/CIT C.jpg]

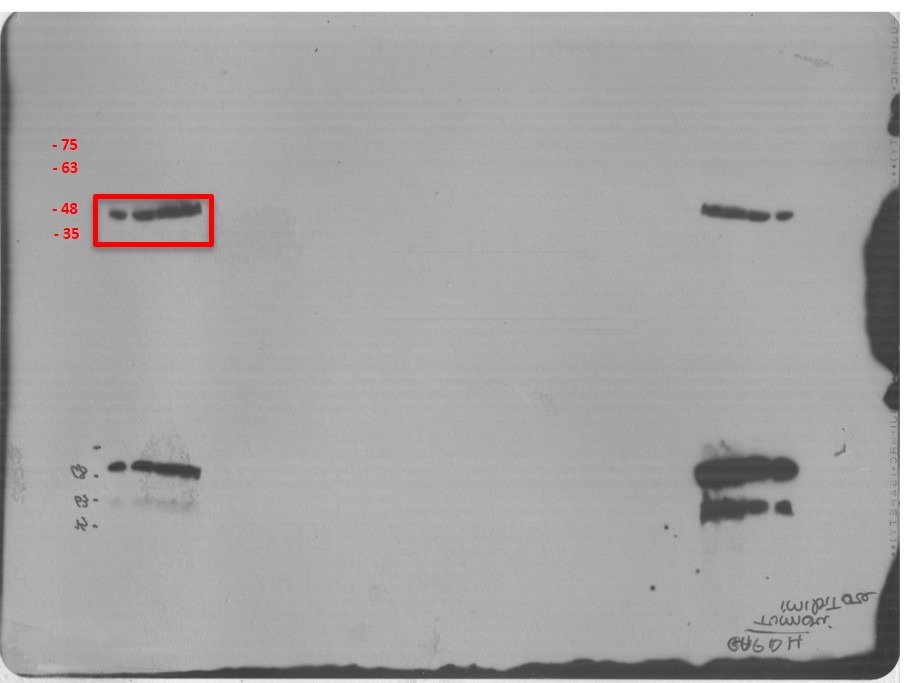

Supplement: Supplementary file 1 [file cancers-12-03530-s001.zip › cancers-1016979-Supplementary Materials/BLOTS/FIGURE 6/PANEL A/ACTINA.jpg]

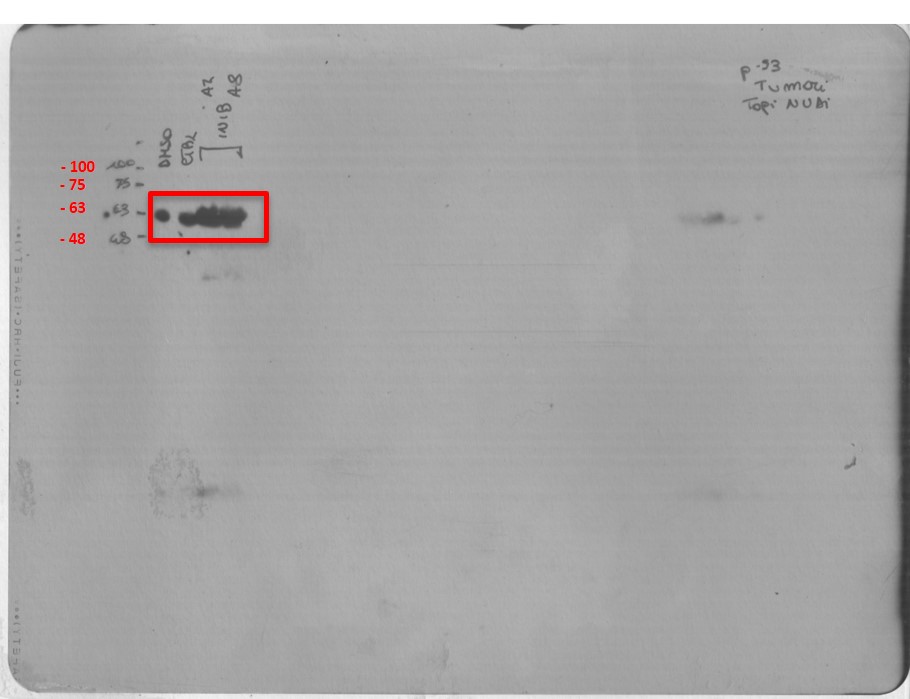

Supplement: Supplementary file 1 [file cancers-12-03530-s001.zip › cancers-1016979-Supplementary Materials/BLOTS/FIGURE 6/PANEL A/P53.jpg]

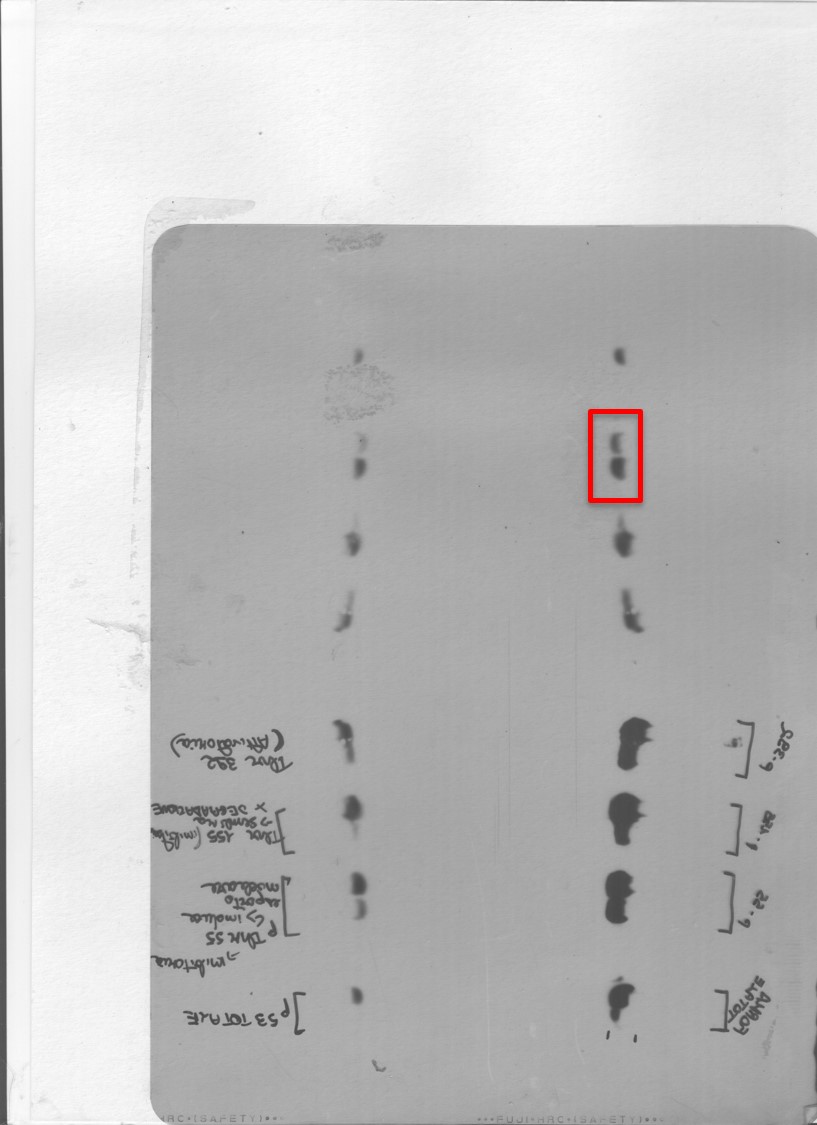

Supplement: Supplementary file 1 [file cancers-12-03530-s001.zip › cancers-1016979-Supplementary Materials/BLOTS/FIGURE 6/PANEL B/P-P53.jpg]

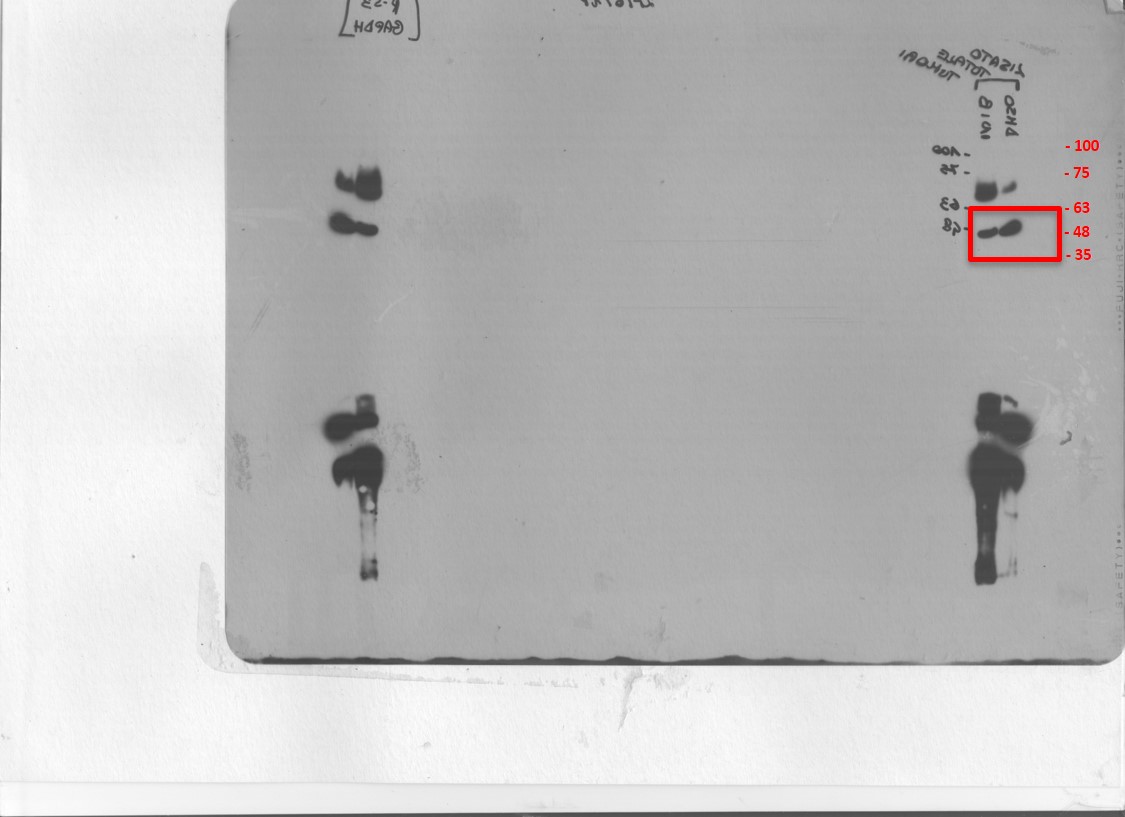

Supplement: Supplementary file 1 [file cancers-12-03530-s001.zip › cancers-1016979-Supplementary Materials/BLOTS/FIGURE 6/PANEL B/P53.jpg]

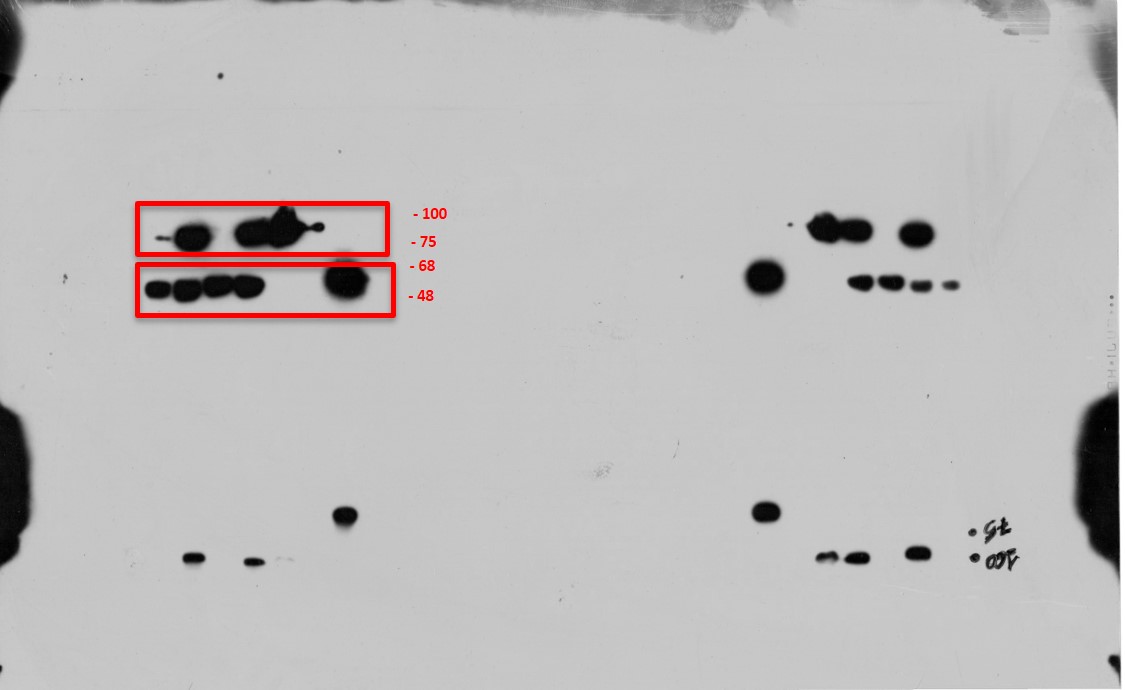

Supplement: Supplementary file 1 [file cancers-12-03530-s001.zip › cancers-1016979-Supplementary Materials/BLOTS/FIGURE 6/PANEL C/IP SERCA P53‬.jpg]

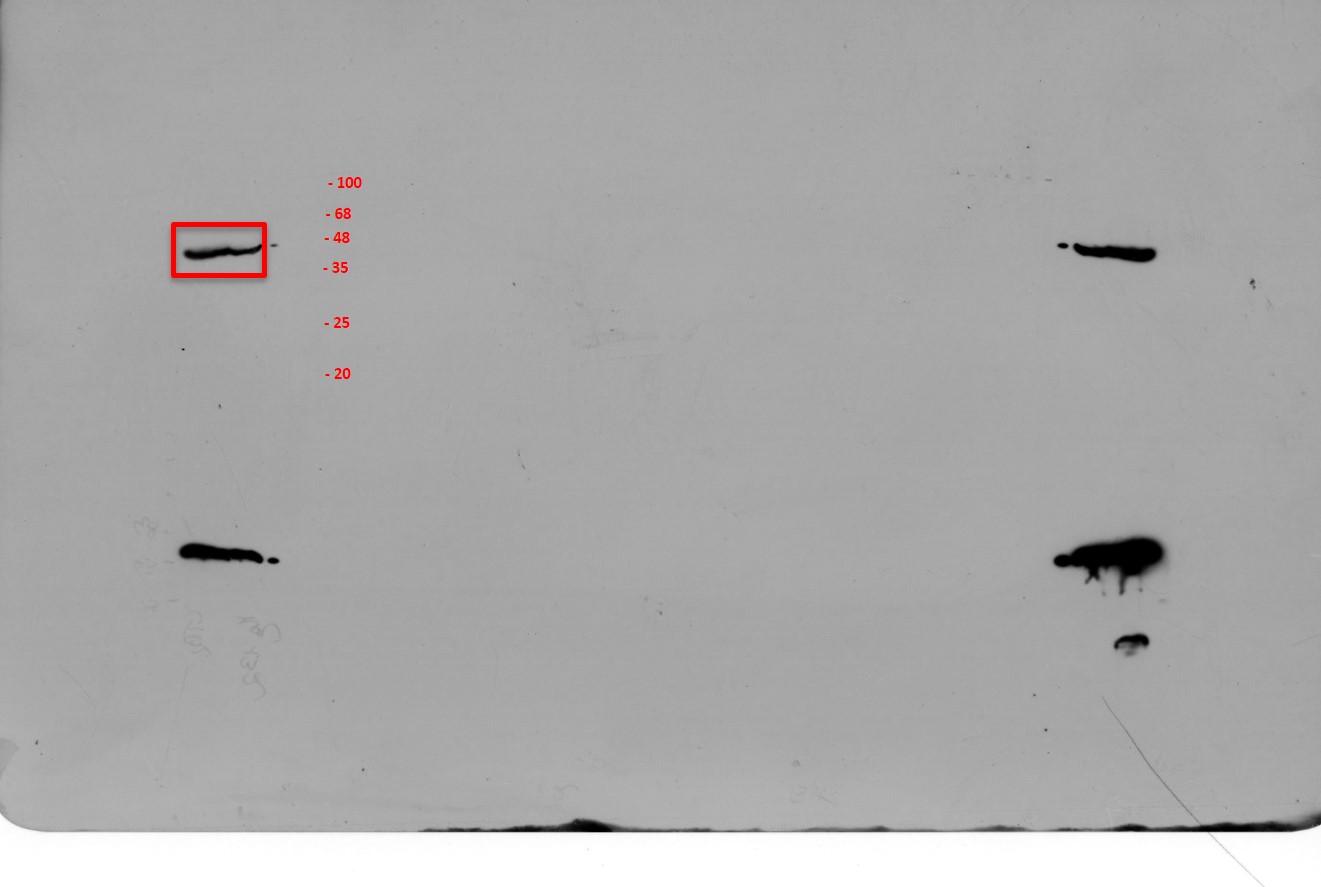

Supplement: Supplementary file 1 [file cancers-12-03530-s001.zip › cancers-1016979-Supplementary Materials/BLOTS/FIGURE 6/PANEL D/ACTIN.jpg]

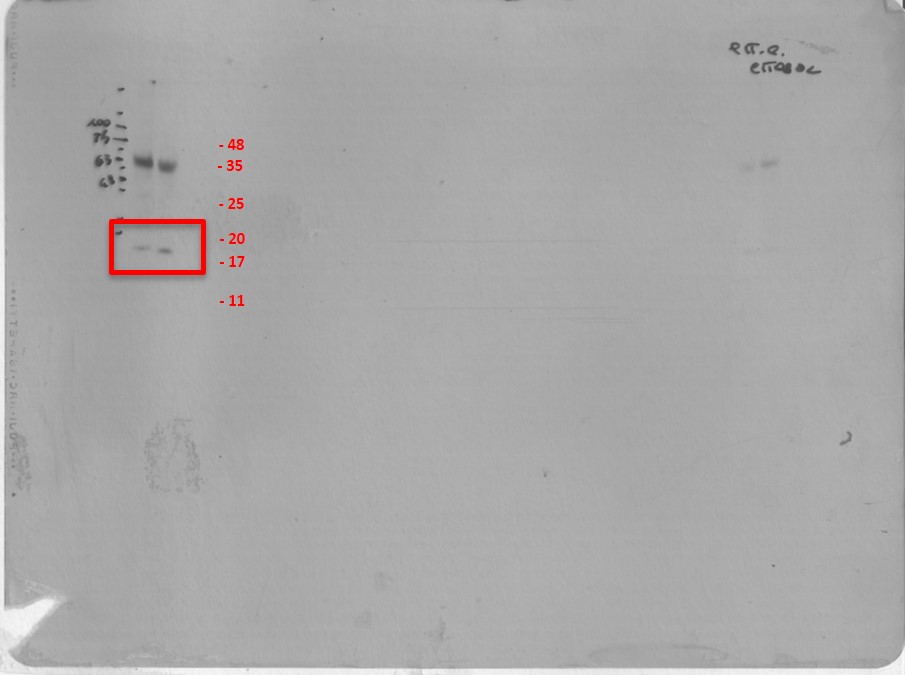

Supplement: Supplementary file 1 [file cancers-12-03530-s001.zip › cancers-1016979-Supplementary Materials/BLOTS/FIGURE 6/PANEL D/CIT C.jpg]
